# Artificial Intelligence Resources for the Screening of Titles and Abstracts in Systematic Reviews: A Scoping Review

**DOI:** 10.1002/cesm.70098

**Published:** 2026-08-14

**Authors:** Ana M. Barragán, Sara Elena Ortiz Bonett, Eliana‐Isabel Rodríguez‐Grande, Alvaro David Orjuela‐Cañón, Oscar J. Perdomo, Guillermo Sánchez‐Vanegas

**Affiliations:** ^1^ School of Medicine and Health Sciences, Public Health Research Group Universidad del Rosario Bogotá Colombia; ^2^ School of Medicine and Health Sciences Universidad del Rosario Bogotá Colombia; ^3^ Universidad Nacional de Colombia Bogotá Colombia; ^4^ Hospital Universitario Mayor Mederi Bogotá Colombia

**Keywords:** artificial Intelligence, automation, screening, systematic review methodology, validation

## Abstract

**Introduction:**

Artificial intelligence (AI) is a branch of technology enabling machines to emulate complex human skills; it can also entail problem‐solving using bioinspired methods. It is used for automating systematic literature reviews (SLR), that is, defining a clinical question, locating relevant literature, preliminary screening, study evaluation, data extraction and analysis. Title and abstract screening is one of the most time‐consuming and error‐prone phases involved in developing a systematic review. While AI promises to expedite this process, adopting it faces challenges due to concerns about compatibility and transparency. This review aims to identify current evidence concerning AI use during preliminary SLR reference screening; it describes characteristics such as the different metrics used for reporting performance and how the different algorithms, pipelines, workflows or web applications are validated. AI resource users' reflections regarding SLR screening automation have also been summarized.

**Methods:**

A scoping review was conducted following Joanna Briggs Institute's (JBI) methodology. Its objective was to identify existing evidence regarding the use of AI resources for title and abstract screening automation. Searches were limited to articles published between 2019 and 2026. The review included primary studies reporting the development, assessment, validation, or real‐world use of AI resources for screening automation, as well as systematic reviews and articles reporting experiences or recommendations for their use. Two types of data were extracted: (1) from primary studies—characteristics of AI resources and, where applicable, recommendations for their use; (2) from systematic reviews and experience‐based articles, recommendations for the use of AI resources. Results included frequency descriptions, tables, figures, and a decision flowchart reflecting the number of references and articles retrieved, excluded, or included in the final analysis.

**Results:**

A total of 174 unique studies published between 2019 and 2026 were included in this scoping review. These were grouped into web applications (43%), model comparisons (32%), generative models (6%), pre‐trained models (3%) or pipelines/workflows (15%) used for title and abstract screening in systematic literature review (SLR). Most studies came from North America. Evaluating these tools often relied on retrospective comparisons with human reviewers' work (63%), sensitivity (*n* = 60), and specificity (*n* = 62) being the most reported metric for criterion assessment and Work Saved over Sampling (*n* = 28) being the most reported metric for assessing their utility. Considerations concerning AI resource use focused on the need for standardized evaluation metrics, stopping criteria, study design and the data sets used, resource characteristics facilitating usability, best practice and future research areas, with the persistence of the human component in the process (*n* = 26) being the most pressing recommendation.

**Conclusion:**

The findings indicated substantial heterogeneity regarding the types of AI resources used, considerable variation concerning the metrics used for reporting performance, differences in how such metrics are defined and a clear need for standardizing reporting methods, study designs and related procedures. Although AI technologies will continue to evolve, maintaining a clear and consistent framework for interpreting research on AI resources for automating title and abstract screening can support understanding their level of maturity and facilitate informed decision‐making by users.

## 1 Introduction

Artificial intelligence (AI) is a technology that enables machines to imitate various complex human skills, including reasoning, learning, problem‐solving, understanding natural language, pattern recognition [1, 2] and, in some cases, problem‐solving using bio‐inspired approaches such as the collective intelligence observed in biological populations. This definition provides a starting point, but is by no means comprehensive, and the field itself is still developing. The main areas of study within the AI field are usually differentiated into natural language processing (NLP), data mining (DM), and machine learning (ML). NLP focuses on enabling machines to understand human language by modeling its syntax, semantics and grammar. Data mining (DM) deals with transforming unstructured data into organized information, whilst machine learning (ML) studies how machines acquire knowledge from identifying statistical patterns and using mathematical or vector representations for decision‐making [3].

One of the applications of AI is the automation of the systematic literature review (SLR). AI is used to free up time, cut costs, and save hours. SLR consisting of several steps, i.e. defining a clinical question and study selection criteria, locating and selecting the relevant literature, evaluating the pertinent literature's quality and risk of bias, extracting data from the included studies, analyzing and presenting the results and interpreting such results.

Title and abstract screening is defined as selecting potential documents which are worth including in the full‐text analysis; this is considered one of the five most time‐consuming SLR steps [4]. A moderate‐sized systematic review, which begins with the title and abstract screening phase, involving around 1600 studies, usually ends up with only 10 to 15 studies being selected; this means that 1000 to 70 000 references are screened by at least two independent reviewers for identifying potential articles according to the defined selection criteria [5]. The time each reviewer spends per reference may be more than 60 s, representing a significant amount of time when multiplied by the amount of references to be screened [6].

Several strategies have been developed for accelerating reference screening; some services operate entirely on human resources [7], whilst others use the human‐input‐then‐AI approach [8] or simply AI for minimizing the number of references reviewed in parallel by using predictive AI as a second reviewer or to triage references in a structured workflow [8, 9]. Using AI for such purpose has led to a split of opinion; the International Collaboration for Automation in Systematic Reviews (ICASR) has made recommendations aimed at incentivising SLR automation [9], the Cochrane Methods (Artificial Intelligence) group, forming part of the ICASR, has been actively engaged in using AI for automating systematic reviews over the last 10 years [10]. By contrast, some leading worldwide clinical practice guideline developers have expressed concern about whether new technologies can be suitably integrated in a way that supports the quality and transparency which have long underpinned current approaches [11]. As SLRs form the best available evidence pillar regarding evidence‐based medicine (EBM), such evidence must be guaranteed to accomplish quality standards, especially showing that such process is transparent and detailed [12].

Articles regarding AI adoption and reporting practices are scarce. Scotti et al., assessed 2271 systematic reviews published between 2017 and 2024 in the Cochrane Database of Systematic Reviews and the Campbell Systematic Reviews and Environmental Evidence journals. The primary findings indicated a tendency for ML use to be concentrated in the screening domain, although it should be noted that only a small percentage of the included studies explicitly reported ML use (12%), as a significant percentage (90%) provided no clarification regarding its use [13]. Barriers included a lack of available user‐friendly software, limited commercialization and scepticism regarding reliability and process guarantees [11]. Some complex AI resources have often been considered “black boxes” due to their poorly‐defined decision‐making processes; such lack of transparency can hinder trust, reproducibility and acceptance [14].

AI use for the automatic selection of primary studies requires extracting features for characterizing such papers and training a classifier to differentiate between those to be included or excluded from SLRs. Features can be extracted using technologies such as NLP and text mining; ML is used for classification where the algorithm learns from a labeled sample of studies, thereby enabling a particular model to predict the relevance of new papers regarding a stated SLR topic [15]. A combination of multiple elements, such as the type of text being processed, the technology used to extract features or a classifier's training approach, creates complex scenarios where no clear consensus exists on how to evaluate [16], validate [17], or report automation outcome performance [18]. Such lack of agreement opens up a potential field of research aimed at clarifying which metrics are the most relevant for the SLR community [19]. Furthermore, using technology/AI‐based SLRs introduces practical challenges affecting transparency and reproducibility. To ensure that others can replicate the process, researchers must clearly document key implementation details, such as the software version used, which packages were installed, data‐cleaning procedures and each step followed throughout the stated workflow.

Since text‐classification resources are built on diverse algorithms, tools and pipelines, they are collectively referred to in this article as AI resources, thereby encompassing all such systems rather than a specific group. Such resources may take the form of single algorithms, integrated systems consisting of several algorithms, or hybrid approaches combining differing technologies.

Although several SLRs have examined resources designed to automate parts of the SLR process, none have specifically focused on how such tools are evaluated, validated or how their performance is measured. Our group thus conducted a scoping review to address this gap, following Joanna Briggs Institute's (JBI) methodology [20]; the concept was defined as the use of AI resources for title and abstract screening, the context as the characteristics of the algorithms used and the outcomes as the performance metrics reported after automation.

### 1.1 Review Question

What is the evidence regarding the characteristics, the performance, validation and/or evaluation metrics used and the methodological considerations related to the use or development of AI resources for automating the reference screening phase of systematic literature reviews' titles and abstracts?

## 2 Materials and Methods

The research question followed the JBI‐recommended Population–Concept–Context (PCC) framework. The Population in this review refers to studies describing how SLRs are conducted or developed including primary studies reporting the development, assessment, validation, or real‐world use of AI resources for screening automation, as well as systematic reviews and articles reporting experiences or recommendations for their use. The Concept focuses on AI resource use regarding how the use or development of partially or fully‐automating initial screening can be based on scoping titles and abstracts, and which are the most frequently used metrics for assessing performance, validation and/or evaluation of such AI resources. Authors' methodological and practical experiences are also recounted, along with their insights drawn from using such AI resources. The Context refers to data sets' fields of knowledge as used for training or testing and the corresponding authors' countries of origin.

### 2.1 Search Strategy

The medical publication databases used included PubMed, EMBASE, SCOPUS, Web of Science, Cochrane, Epistemonikos and technology‐related databases, such as IEEE Xplore and Science Direct; Google Scholar was used for finding some grey literature, in addition to a manual identification article. Controlled language terms and free text were used to build the master strategy in MEDLINE (Universidad del Rosario); this search strategy was then adapted for other databases. All searches were limited to studies published between 2019 and the end of February 2026. Such recent 6‐year period was focused on because technology in this area has evolved extremely quickly, involving frequent software updates and methodological advances. In particular, the field of NLP‐related AI has changed dramatically after the public release of ChatGPT in 2022, built on the 2017 Transformer architecture that reshaped how machines understand and generate human language [21]. Systematic review search terms were included solely to capture reports of AI‐assisted screening automation occurring within the systematic review process; the unit of analysis remains the AI resource or its recommended use, not the reviews themselves. A total of seven studies were included through manual search, identified through reference tracking and a targeted search for tools currently participating in the Cochrane Innovative Platform Study [22]. Appendix 1 contains all search strategies used, ending in February 2026.

### 2.2 Study Selection

All searches were uploaded to the Rayyan web‐based research collaboration platform [23], using the free version's functionalities (duplicates were removed). AI was not used for screening or for the rest of the scoping review. Titles and abstracts were screened manually by at least two independent reviewers (A. M., E. R., N. B., L. C., S. O., G. G., L. V., L. O., L. V., and S. M.) who attempted to exclude obvious non‐relevant references. All references were screened by humans; all reviewers followed a hierarchical screening tool's guideline, with clearly put questions to guide their judgment. All disagreements were resolved by consensus. Potentially relevant papers' full texts were retrieved.

**Figure 1:**
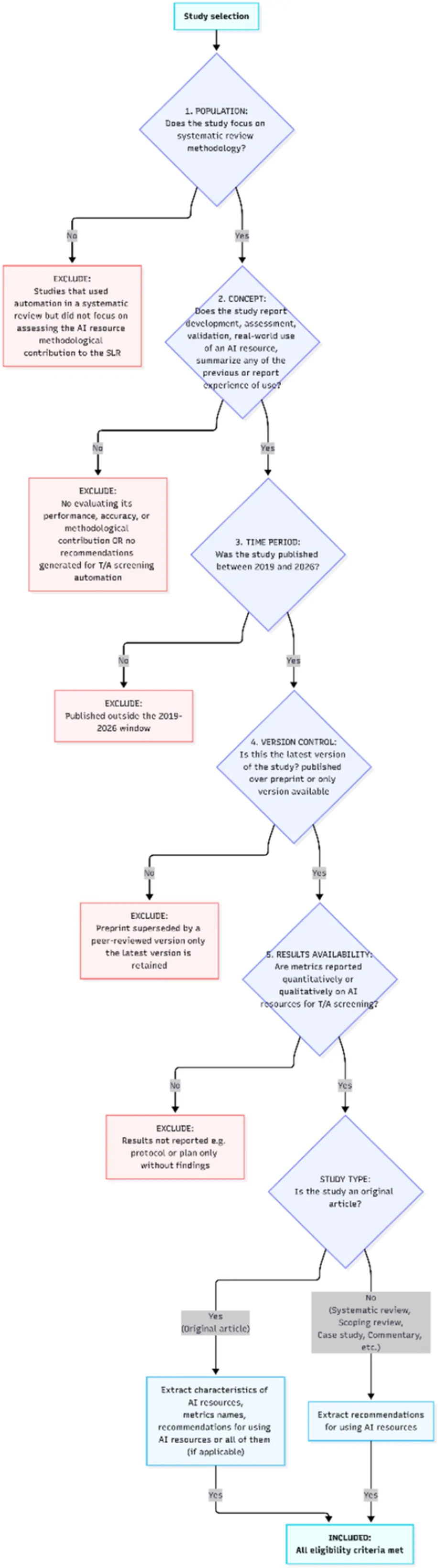
Flowchart of inclusion and exclusion criteria AI resources for the screening of titles and abstracts in systematic reviews. Figure code generated using Claude 4.6 Sonnet and rendered via Mermaid. Final layout edited and verified by the authors.

The review included articles describing systematic review methodologies that incorporated AI resources for automating title and abstract screening, including studies concerning the development or evaluation of these resources, as well as secondary research summarizing AI resources designed to fully or partially support this screening phase (Figure 1. Flowchart of inclusion and exclusion criteria regarding AI resources for the screening of titles and abstracts in systematic reviews).

### 2.3 Data Extraction

Spreadsheet forms were created which were piloted and adjusted before their use. The whole data extraction team was trained regarding definitions and criteria for filling the matrix. At least two extraction team members collected data independently and in parallel for each variable.

The data extraction form included basic information about each article, such as the year of publication, the first author's surname and corresponding author's stated country of origin. A separate instrument was used for collecting data regarding the type of AI resource described, the authors' purpose in using the AI resource, whether the authors reported a comparator, the metrics used for presenting outcomes after comparison and the types of outcomes reported. We extracted data using two instruments, each applied to different types of studies. From primary studies, we extracted characteristics of the AI resources and the names of the metrics reported, among others. A second instrument captured variables related to authors' considerations and reflections regarding the use of AI resources, which was applied to studies reporting experiences, recommendations, or secondary research. Appendix S2 provides a detailed description of the exclusions at this phase.

### 2.4 Data Analysis and Presentation

Descriptive statistics were used for summarizing the extracted characteristics. A narrative summary was made regarding the extracted information, figures, and tables.

An iterative analysis was made of the collected variables, interpreting the sets of documents within their contexts and creating thematic categories making sense of the data so collected; this was necessary for synthesizing the information due to the heterogeneity of concepts, and authors' definitions regarding the metrics used. No risk of bias (RoB) assessment was made. The protocol was registered and is available at: https://osf.io/yzsr8/.

**Figure 2:**
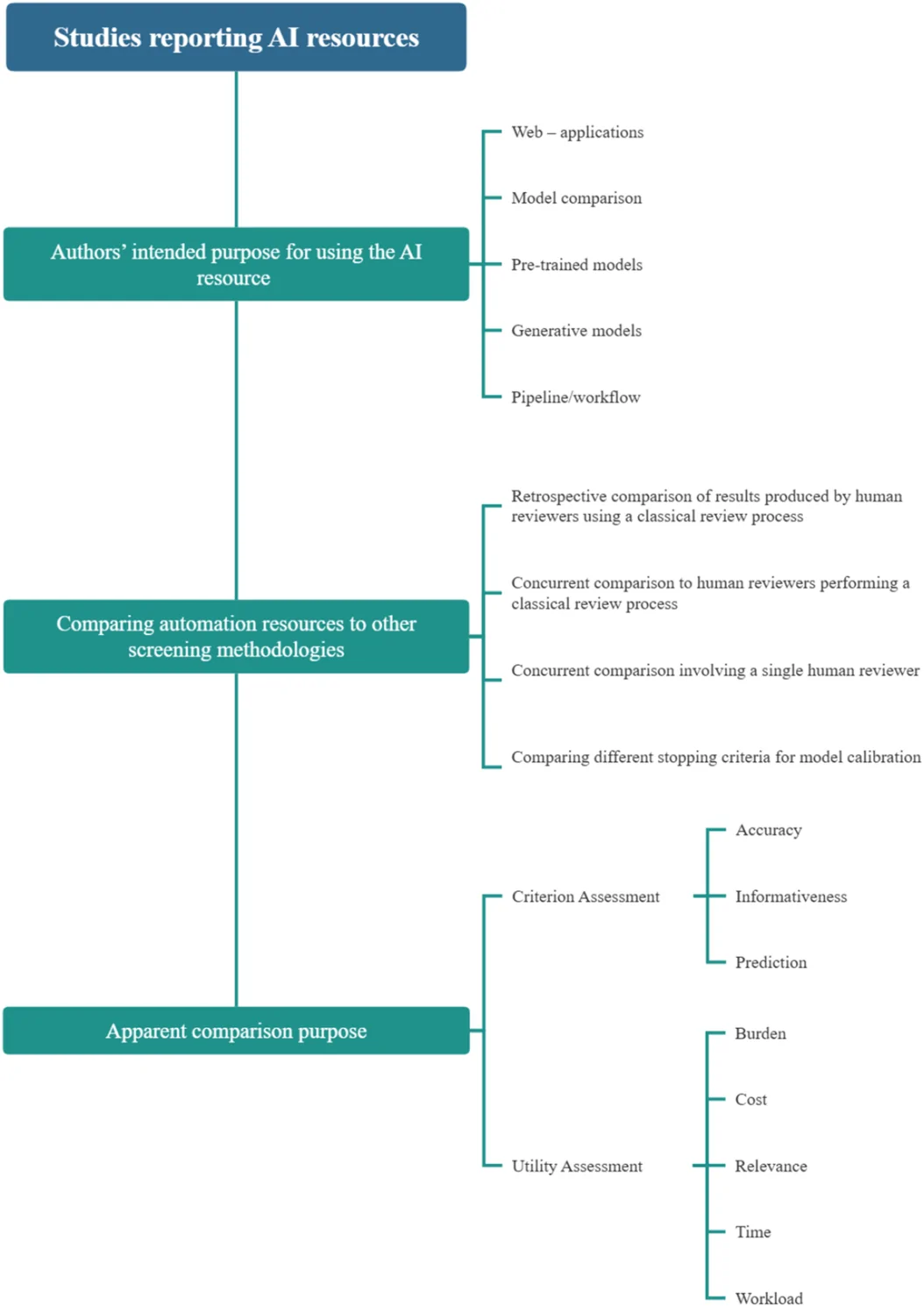
Diagram of variables extracted from studies reporting AI resource use (*Source:* Authors' elaboration). Figure created using GitMind. Authors development.

**Figure 3:**
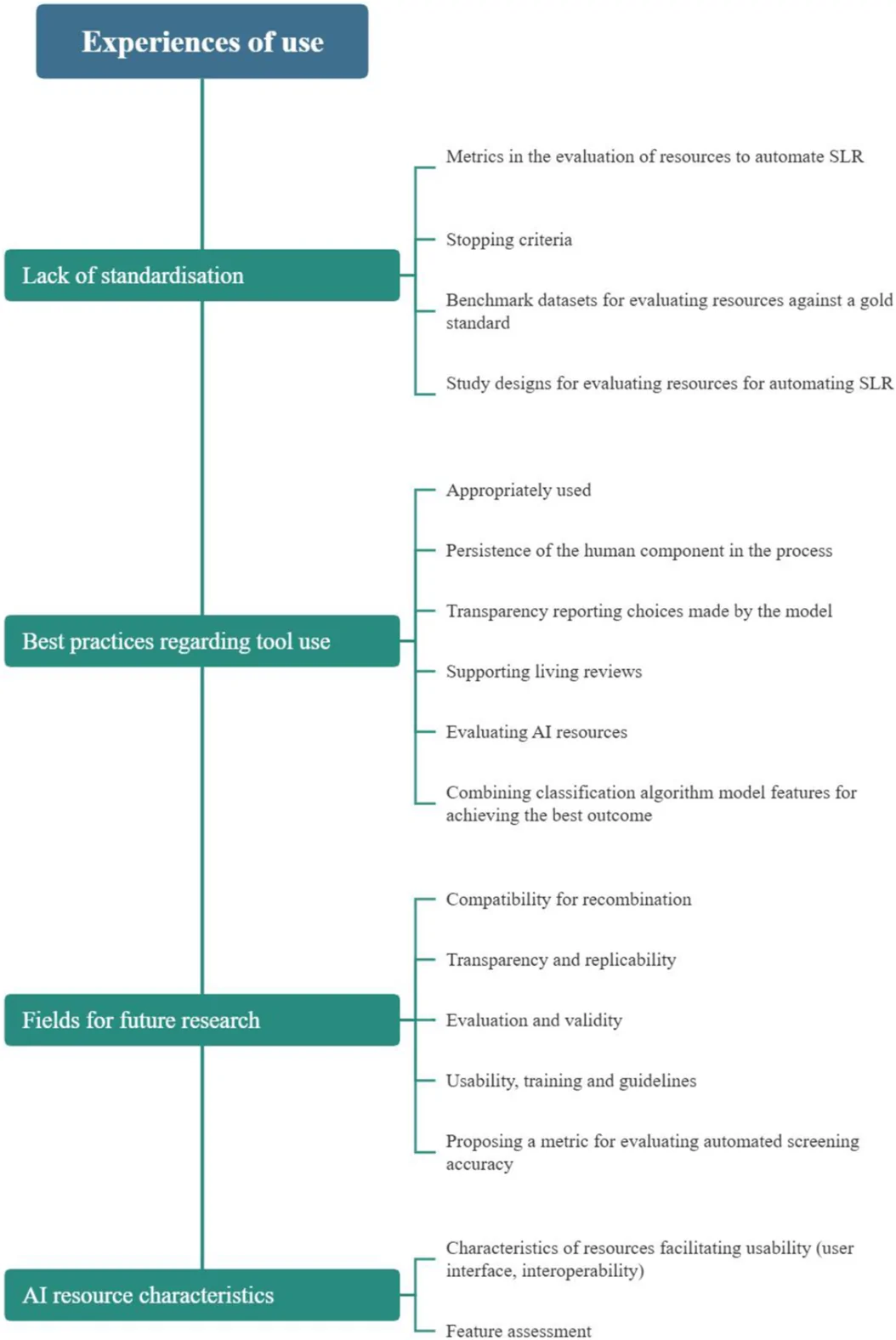
Diagram of variables extracted from studies reporting experience regarding the use of automation tools. (*Source:* Authors' elaboration). Figure created using GitMind. Authors development.

Multiple categories and subcategories were created, building on psychometry and epidemiology concepts to ensure conceptual coherence and interpretability within a health research context. The definition of validation has been adopted in this article as the process of accumulating empirical evidence to support the interpretation of the metrics reported in the retrieved studies [24, 25, 26]. First, broad categories were defined for organizing the extracted information according to major analytical domains; subcategories or third level categories were then created within each to provide finer distinctions (Figure 2. Diagram of variables extracted from studies reporting AI resource use—Figure 3. Diagram of variables extracted from studies reporting experience regarding the use of automation tools).

Regarding the characteristics of AI resources, five main categories were defined to group them based on our interpretation of each article's intent, focusing on the scope of the results rather than strictly on the technological classification. The first grouped Web applications where the main results focused on describing a complete software system with a user interface, either installed locally on a device or hosted in the cloud and accessed via URL. Second, the model comparison category grouped articles reporting model training or machine learning (ML), deep learning (DL) or large language model (LLM) fine‐tuning. The third group of Pre‐trained models referred to articles mainly reporting fine‐tuning a pre‐trained model or describing methods used for adjusting features weighting the algorithm used mainly BERT and variants. Fourth, the generative models' category included articles reporting AI resources based on GPT that were not used through interfaces installed locally on a device or accessed via cloud‐based platforms such as ChatGPT. Finally, the fifth, pipelines/workflows category grouped AI resources combining different technologies for the execution of specific steps, such as training, inference or excluding conventional steps. It is possible that an article reporting only generative AI resources could be classified under model comparison, as the authors' intent may have been to compare different versions of resources within the same technology

Data was extracted concerning information related to comparing automated AI resources with any other screening methodology. All pertinent reported metrics were extracted and summarized by overall study frequency; two subcategories were defined within this category. One was defined as “Criterion assessment” because the authors aimed at reporting AI resources' level of discrimination or predictive accuracy, as well as the degree of agreement compared to other screening methodologies. This category included contingency table‐derived metrics [25, 27].

The metrics were further classified according to their analytical domain. Third‐level categories included *accuracy* for metrics showing how accurately a classifier had captured each reference's true status. *Prediction* related how well AI automation output reflected a reference's actual relevance. Metrics indicating changes regarding the probability of relevance were classified as *informativeness*. Specific metric values were not extracted due to the large volume of data, as many articles reported multiple computational experiments.

The second category, defined as “Utility assessment,” referred to outcomes which evaluated research teams' added value, particularly their ability to identify relevant references more efficiently or at a lower cost compared to manual screening. Metrics were further classified within this category according to their specific purpose, resulting in five subcategories: *burden, cost, relevance, time*, and *workload*. Regarding such diversity of metrics, authors' definitions were collected and organized into corresponding categories.

Along with extracting metrics, the authors' reflections and recommendations concerning the use of AI resources in the screening process were also analyzed. Direct quotes were extracted from the included articles and put into five thematic categories, defined according to the scope of the authors' recommendations.Those addressing AI resources' intrinsic and methodological aspects were labeled *AI resource characteristics. Standardization and metrics* grouped statements calling for the need to standardize thresholds, definitions and/or validation methodologies for improving AI resources' comparability. *Best practices regarding tool use* focused on their practical application and advised how to use a particular tool appropriately, in which circumstances and with the necessary precautions. *Fields for future research* captured forward‐looking perspectives and areas identified for further exploration.

Data visualizations were generated using AI‐assisted tools to optimize graphical representation. Code synthesis was performed via AI models, while graphical rendering was executed within standard programming environments, as explicitly documented in the respective figure footnotes. The authors maintained full control overall raw data and data processing stages, conducted comprehensive quality control procedures, and independently validated the final visual outputs. The underlying data sets and code are available from the authors upon reasonable request.

## 3 Results

### 3.1 Study Inclusion

**Figure 4:**
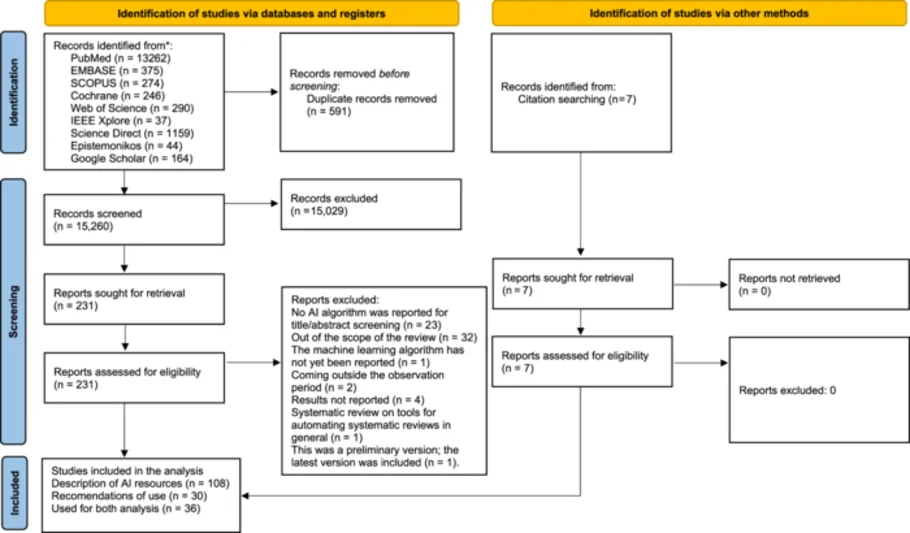
Search conducted from November 2023 to February 2025, which was updated in February 2026. (*Source:* Page M. J., et al. *BMJ* 2021;372:n71. doi: 10.1136/bmj.n71). This work is licensed under CC BY 4.0. To view a copy of this license, visit https://creativecommons.org/licenses/by/4.0/.

Database searches led to identifying 15 851 records and manual identification led to 7 records, after removing duplicates, 15 267 records were screened for relevance; 231 articles were retrieved for full text review. A total of 174 unique studies were included in this scoping review. Of these, 108 studies described AI resources; within this group, 36 studies also reported recommendations for using AI resources. An additional 30 studies were included solely for recommendations for using AI resources, without describing any AI resource. A decision flowchart was constructed to show the number of references and articles retrieved, excluded from or included in the final analysis (Figure 4. Search conducted from November 2023 to February 2026).

### 3.2 General Characteristics Regarding Included Studies

**Figure 5:**
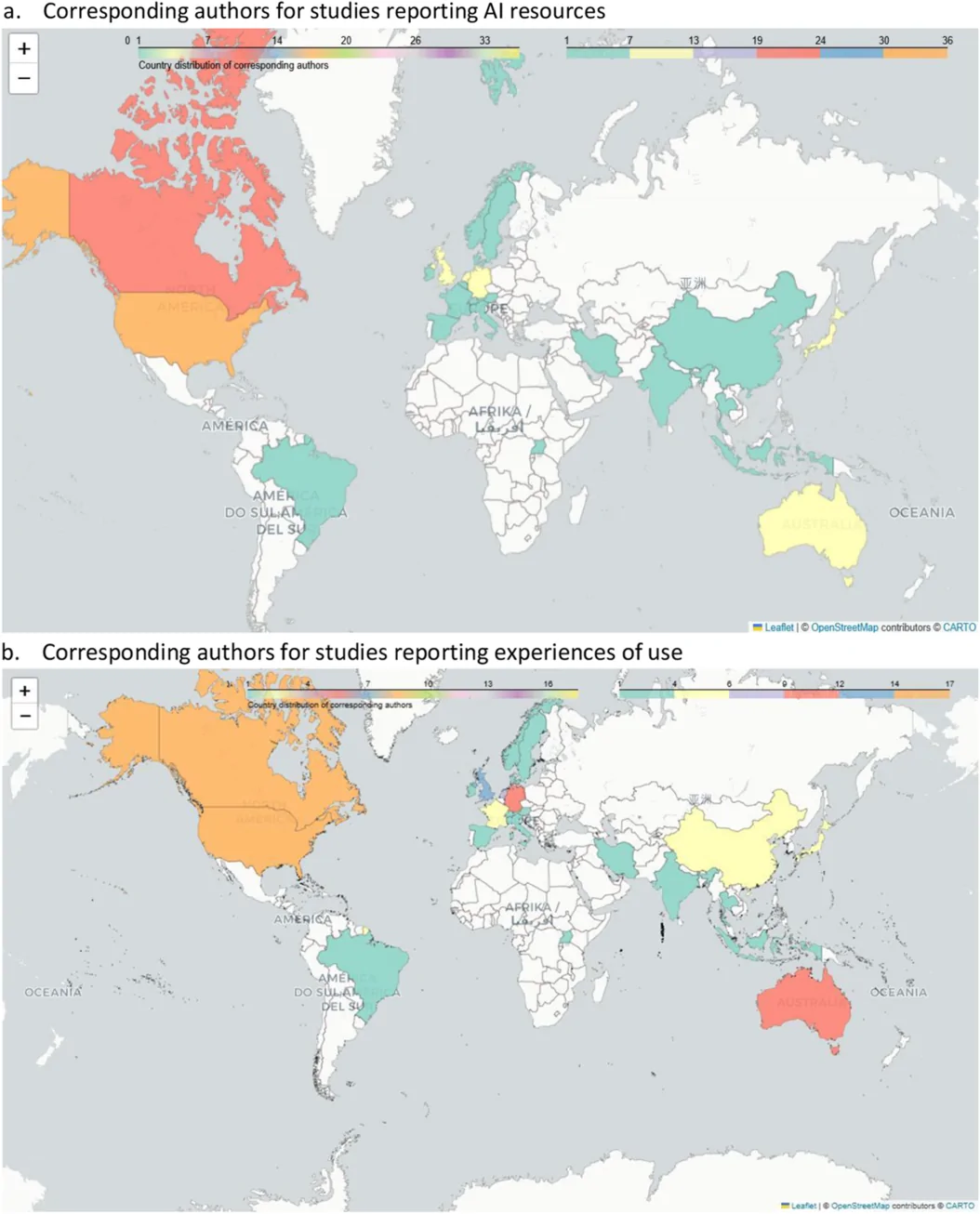
Map showing the countries of corresponding authors' stated affiliations.(*Source:* Authors' elaboration). (a) Corresponding authors for studies reporting AI resources. Figure code generated using Claude 4.6 Sonnet and rendered via Antigravity. Final layout edited and verified by the authors. Descriptive statistics showing the frequency of corresponding authors' affiliations for studies reporting AI resources as an indicator of the AI resources' publication patterns regarding automated screening in systematic reviews. (b) Corresponding authors for studies reporting experiences of use. Figure code generated using Claude 4.6 Sonnet and rendered via Antigravity. Final layout edited and verified by the authors. Descriptive statistics showing the frequency of corresponding authors' affiliations for studies reporting experiences of use as an indicator of the AI resources' publication patterns regarding automated screening in systematic reviews.

The year in which most publications were reported was 2025 (*n* = 62 articles) [28, 29, 30, 31, 32, 33, 34, 35, 36, 37, 38, 39, 40, 41, 42, 43, 44, 45, 46, 47, 48, 49, 50, 51, 52, 53, 54, 55, 56, 57, 58, 59, 60, 61, 62, 63, 64, 65, 66, 67, 68, 69, 70, 71, 72, 73, 74, 75, 76, 77, 78, 79, 80, 81, 82, 83, 84, 85, 86, 87, 88, 89]. Relevant publications were found from 29 countries, the countries having the most publications being the United States of America (*n* = 44) [19, 29, 31, 34, 35, 43, 44, 45, 48, 49, 51, 54, 57, 65, 66, 67, 69, 70, 71, 77, 82, 84, 87, 88, 89, 90, 91, 92, 93, 94, 95, 96, 97, 98, 99, 100, 101, 102, 103, 104, 105, 106, 107, 108], followed by Canada (*n* = 20) [9, 30, 61, 64, 72, 79, 109, 110, 111, 112, 113, 114, 115, 116, 117, 118, 119, 120, 121, 122] (Figure 5, Map showing the countries of corresponding authors' stated affiliations).

The majority of studies (41%) used a unique data set. The median number of databases per study was 2.0 (25th percentile Q1: 1.0; 75th percentile Q3: 4.0). The median dataset size was 1880 references (Q1: 684; Q3: 5043). We explored the distribution of sample sizes by the number of data sets used per study and by publication period. As no statistical tests were performed, these exploratory visual inspections are presented for descriptive purposes only (Appendix S3, Overview and temporal trends of data sets and AI resource use in screening studies (2019–2026)). Seventy‐eight percent of the included articles used existing tools, while the remaining studies presented AI resources developed by the authors. The full characteristics are presented in Table 1(a), reporting the use of AI resources for automation screening, and Table 1(b) reporting experiences regarding the use of automation AI resources.

**Table 1:** Characteristics of studies included in the analysis.

a. Evidence regarding performance and utility					
References	First author's surname	Correspondence author affiliation	Year of publication	AI resource reported (information from the best resource reported by the authors was extracted)	Dataset subject area
[123]	van Dinter	Qatar	2021	Decision support system (DSS)	Bisphenol‐A and obesity, PFOA/PFOS immunotoxicity, Transgenerational inheritance of health effects, Fluoride and neurotoxicity in animal models, Neuropathic pain
[4]	Clark	Australia	2020	RobotSearch, SRAhelper	Urinary tract infection interventions (RCT)
[124]	Carey	Ireland	2022	Abstrackr	Diffuse large B‐cell lymphoma
[125]	Bravo	Spain	2021	Logistic regression (LR), support vector machines, stochastic gradient descent	Pancreatic cancer, Indoor allergen reduction, Inguinal hernia
[111]	Popoff	Canada	2020	Support vector machine (SVM), Naïve Bayes, bagged CART	Liver cancer, Lung cancer, Psoriasis, Melanoma, Obesity
[112]	Hamel	Canada	2020	DistillerSR	Hot flashes, Opioid use disorder, Meniere´s disease, Nonsmall cell lung cancer, Prophylaxis for influenza, Smoking cessation, Asthma/urticaria, Depression screening, Prophylaxis for HIV, Sugar sweetened beverages
[126]	van den Bulk	The Netherlands	2022	Logistic regression (LR), support vector machine (SVM), Naive Bayes (NB), random forest (RF), AdaBoost (AB), Gradient boosting (GB), long short‐term memory (LSTM), bidirectional encoder representations from transformers (BERT)	Leafy greens, Cereals
[8]	Noel‐Storr	UK	2020	Randomized controlled trial (RCT) classifier	Antidepressant benefits and harms, Psychological, complementary, and exercise treatment for major depression
[97]	Reddy	USA	2020	RobotAnalyst, Abstrackr	Early‐stage prostate cancer treatments
[97]	Gates	Canada	2020	Abstrackr	Concussion, Depression safety, Depression treatments, Diabetes, Digital technologies for pain, Experiences of bronchiolitis, Experiences of UTIs, Preterm delivery, Treatments for bronchiolitis, VBAC, Visual acuity, Workplace stress
[127]	Yamada	UK	2020	Concept Encoder	Diabetes, Cardiology, Cerebral haemorrhage
[128]	Giummarra	Australia	2019	Abstrackr	Injury‐focused systematic reviews
[129]	Tsubota	Japan	2022	Bidirectional encoder representations from transformers (PubMedBERT)	EBM‐NLP: publicly available corpus comprising 5,000 annotated abstracts from articles describing clinical randomized controlled trials
[98]	Zimmerman	USA	2021	SVM with stochastic gradient decent, K‐nearest neighbors (KNN), decision trees, Sigmoidal SVM	Diabetes
[130]	Lange	Germany	2021	Naive Bayes, L2‐Logistic regression, C5‐0, random forest (RF), SVM, multilayer perceptron, convolutional neural network, soft voting, stacking	Orthopaedics
[99]	Tsou	USA	2020	Abstrackr, EPPI‐Reviewer	Pancreatic cancer, Indoor allergen reduction, Inguinal hernia, Dabigatran, TAVI, Bronchial thermoplasty, Digital tomosynthesis, Faecal transplantation for *clostridium difficile*, Intragastric balloon
[100]	Lee	USA	2020	Multimodal Missing Data aware Stacked Autoencoder (MMiDaS‐AE)	ACE inhibitors, ADHD, Antihistamines, Atypical antipsychotics, Beta blockers, Calcium channel blocker, Oestrogens, NSAIDs, Opioids, Oral Hypoglycaemics, Proton pump inhibitors, Skeletal muscle relaxants, Statins, Triptans, Urinary incontinence, Anaemia, COPD, Clopidogrel, Proton beam
[131]	Qin	China	2021	Light gradient boosting machine (LightGBM), BERT	Randomized controlled trials, Sodium‐glucose co‐transporter‐2, Diabetes
[132]	Chai	Australia	2021	Research screener	Sedentary ‐ overweight, Low back pain, Lung cancer, Falls, Acute pain, Sexual health, Back pain education, Ankyloglossia assessment
[133]	Callaghan	UK	2020	Support vector machine (SVM)	Urinary incontinence, Antihistamines, Oestrogens, NSAIDs, Oral Hypoglycaemics, Triptans, ADHD, Atypical antipsychotics, Calcium channel blocker, Proton pump inhibitors, Skeletal muscle relaxants, COPD, Kitchenham, Opioids, Beta blockers, ACE inhibitors, Stains, Proton beam, Radjenovic, Wahono, Hall
[101]	Howard	USA	2020	SWIFT‐Active Screener	PFOA/PFOS and immunotoxicity, BPA and obesity, Transgenerational inheritance of health effects, Fluoride and neurotoxicity in animal models, Neuropathic pain, Skeletal muscle relaxants, Antihistamines, Opioids, ADHD, Triptans, Urinary incontinence, ACE inhibitors, NSAIDs, Beta blockers, Proton pump inhibitors, Oestrogens, Statins, Calcium channel blocker, Oral hypoglycaemics, Atypical antipsychotics, Mammalian
[134]	Prasetyo	Indonesia	2022	Multinomial Naïve Bayes (MNB), support vector machine (SVM), simple dense neural network, long short‐term memory (LSTM), convolutional neural network (Conv‐ID), universal sentence encoder (USE), gated recurrent units (GRU), bidirectional LSTM	Edentulous and comorbid diseases
[115]	Pham	Canada	2021	Random Forest classifier	Diabetes, KS methods
[135]	van Altena	The Netherlands	2021	Random Forest classifier	Tuberculosis, Parasitic infections, Cancer, Dementia, Alzheimer's, Liver, Skeletal muscle system, Down's syndrome
[102]	Ambalavanan	USA	2020	Cascade learner and individual task learner (SciBERT model)	Clinical Hedges dataset
[136]	Van de Schoo	Netherlands	2021	ASReview	Virus metagenomics in farm animals, Software engineering fault prediction, Posttraumatic stress trajectory analysis, Angiotensin‐converting enzyme inhibitors' efficacy
[137]	Kataoka	Japan	2023	Bidirectional encoder representations from transformers (PubMedBERT), BioM Electra, SapBERT	COVID‐19, Malignant pleural effusion, Inflammatory bowel diseases, Bird fancier's lung, Gastric tube placement, Aortic dissection, Glioma, Dementia with Lewy bodies, Cause of fever of unknow origin, Acute meningitis, Pulmonary hypertension, Cardiology studies
[138]	Muthu	India	2023	ASReview	Cell therapy regarding rotator cuff tears, Mesenchymal stem cells in knee osteoarthritis, Spine surgery Fragility outcomes
[116]	Tetzlaff	Canada	2019	Support vector machine (SVM), Naïve Bayes	Health technology, Economics
[139]	dos Reis	Brazil	2023	Rayyan, Abstrackr, Colandr	Musculoskeletal pain management
[140]	Natukunda	Uganda	2023	Latent Dirichlet Allocation (LDA)	Helminth data set, Wilson's disease dataset
[141]	Forsgren	Sweden	2023	EPPI‐Reviewer	Randomized controlled trials
[142]	Olorisade	UK	2019	Support vector machine (SVM)	Drug Evaluation Review Program (DERP) systematic reviews: Kitchenham, Hall, Wahono, Radjenovic, ACE inhibitors, ADHD, Antihistamines, Atypical antipsychotics, Beta blockers, Calcium channel blockers, Oestrogens, NSAIDs, Opioids, Oral Hypoglycaemics, Proton pump inhibitors, Skeletal muscle relaxants, Statins, Triptans, Urinary incontinence
[117]	Saeidmehr	Canada	2024	Logistic regression, Naïve Bayes, support vector machine (SVM), Random Forest	Cognitive behavioral therapy, Anxiety‐related disorder and Posttraumatic stress disorder (PTSD), National culture
[143]	Tran	France	2023	GPT‐3.5	COVID‐19, Pharmacologic treatment epidemiology, Dairy intake regarding intermediate disease markers, Pharmacological treatments for chronic plaque psoriasis
[118]	Li	Canada	2023	Rayyan, Abstrackr, SWIFT‐Review	Pregnancy complications and cardiovascular risk
[144]	Ng	Singapore	2023	Bidirectional encoder representations from transformers (PubMedBERT)	End‐stage lung disease
[145]	Ferdinands	The Netherlands	2023	Naïve Bayes, logistic regression, support vector machine (SVM), Random Forest, term frequency‐inverse document frequency (doc2vec)	Wilson's disease, Posttraumatic stress disorder (PTSD), Software fault prediction, ACE‐inhibitors, Metagenomic sequencing (mNGS)
[146]	Lanera	Italy	2019	Support vector machine (SVM), Random Forest, K‐nearest neighbors (KNN), elastic‐net regularized generalized linear models (GLMs)	Atrial fibrillation, Psoriasis, Colorectal cancer, Gastric cancer, Alzheimer's disease, Parkinson's disease, Diabetes, Rheumatoid arthritis, Hypertension
[119]	Perlman‐Arrow	Canada	2023	PubMed‐BERT, Covidence	SeroTracker's living systematic review database regarding SARS‐CoV‐2 seroprevalence
[16]	Kebede	Germany	2023	Naïve Bayes, support vector machine, regularized logistic regressions, neural networks, Logit boost, XGBoost	Body fatness regarding sex steroid hormone‐related outcomes
[9]	Gates	Canada	2019	Abstrackr, DistillerSR, RobotAnalyst	Antipsychotics, Bronchiolitis, Visual acuity
[147]	Norman	France	2019	Waterloo CAL	Diagnostic test accuracy
[148]	Waffenschmidt	Germany	2023	Rayyan, EPPI‐Reviewer, web‐based Trial Selection DataBase (webTSDB)	Health technology assessments
[149]	Oami	Japan	2023	ASReview	Emergency medicine, Critical care, Anaesthesiology
[150]	Oude Wolcherink	The Netherlands	2023	ASReview	Early detection strategies for cardiovascular disease
[151]	Simon	Denmark	2019	BioReader (Biomedical Research Article Distiller), Medline ranker	Tumor suppressor gene p53
[121]	Guo	Canada	2024	GPT‐4	COVID‐19 treatment and antimalarials, Nonopioid analgesia, Raynaud's syndrome
[152]	Gartlehner	USA/Austria	2019	DistillerSR	Pharmacological and nonpharmacological interventions for treating depression
[153]	Nedelcu	Germany	2023	ASReview	Prostate Imaging‐Reporting and Data System (PI‐RADS) prostate cancer diagnostic performance
[35]	Purewal	USA	2025	ChatGPT‐4	Chronic pain research (emotional functioning after spinal cord stimulation (SCS) implantation)
[154]	Matsui	Japan	2024	GPT‐3.5‐turbo, GPT‐4	Systematic reviews on bipolar disorder treatment (clinical studies in psychiatry: bright light therapy and adjunctive pharmacotherapy)
[155]	Wiwathanasetthakarn	Thailand	2024	Few‐Shot Learning (FSL) model framework with S‐BERT	Therapy, Prognosis/risk, Genetic association, Economic evaluation
[156]	Campos	Norway	2024	Random Forest (RF) with sentence bidirectional encoder representations from transformers (SBERT)	Systematic reviews on education and educational matters
[157]	Gargari	Iran	2024	GPT‐3.5 Turbo	Light therapy regarding insomnia disorder
[158]	Pijls	The Netherlands	2024	ASReview	Ribbing disease, radiostereometric analysis (RSA), Metal‐on‐metal hip arthroplasty
[159]	Menold	Germany	2024	Random Forest, logistic regression with elastic net regularization (LogReg), support vector machine (SVM)	Perioperative administration of blood transfusions during radical cystectomy, Radiomics for kidney tumor classification, Bladder cancer
[160]	Pilz	Germany	2024	Support vector machine (SVM), logistic regression, Random Forest, LightGBM	Interventions to reduce the incidence of surgical site infection in colorectal resections (INTRISSI), Comparison of surgical and alternative approaches for the treatment of perforated peptic ulcers (PPU)
[108]	Chan	USA	2024	ASReview	Dental implant failure and penicillin allergy, Laser therapy and peri‐implantitis, Soft tissue grafting and immediate implants, Implant survival rate of simultaneous GBR, Preeclampsia and periodontitis, Platelet‐rich fibrin (PRF) in intrabony defects
[161]	Issaiy	Iran	2024	ChatGPT‐3.5 Turbo	Diagnostic radiology, Nuclear medicine, Interventional radiology
[162]	Dennstädt	Switzerland	2024	FlanT5‐XXL (FlanT5), OpenHermes‐2.5‐neural‐chat‐7b‐v3‐1‐7B (OHNC), Mixtral‐8 × 7B‐Instruct v0.1 (Mixtral), Platypus2‐70B‐Instruct (Platypus 2)	Radiation oncology
[163]	Khraisha	Ireland	2024	ChatGPT‐4	Parenting in protracted refugee situations
[164]	Akinseloyin	UK	2024	GPT‐3.5 Turbo, LLama 2, gemini, Claude 3	Technology‐assisted reviews in empirical medicine
[107]	Du	USA	2024	Extreme Gradient Boosting (XGBoost), support vector machine, logistic regression, Random Forest, BERT base, BioBERT base, PubMedBERT	Human papillomavirus, Paediatric pneumococcal infection
[165]	Tran	France	2024	GPT‐3.5 Turbo	Dairy intake regarding intermediate disease markers, Pharmacology, COVID‐19
[122]	Burns	Canada	2020	DistillerSR	Anaesthesia
[106]	Li	USA	2024	ChatGPT‐4, ChatGPT‐3.5, Google PaLM 2, MetaLlama 2, ChatGPT‐4‐Turbo, ChatGPT‐3.5‐Turbo, Gemini‐1.0, Llama 3, Claude 3 opus	Animal depression, Rheumatic disease, Environmental health
[18]	König	Germany	2024	ASReview	Applied psychology, Clinical psychology, Developmental psychology, Educational psychology, Social psychology
[166]	Thomas	France	2024	GPT‐3.5‐Turbo	Ecosystem condition, Ecosystem services, Ecosystem accounting
[167]	Sugiura	Japan	2024	Rapid Medical Evidence Synthesis (RMES)	Malignant neoplasms, Heart disease, Cerebrovascular disease, Hypertension, Diabetes, Dietary supplements
[47]	Zdorovtsova	UK	2025	GPT‐3.5‐Turbo, GPT‐4‐Turbo	Randomized‐Controlled Trials (RCTs), Observational studies, Average number of abstracts
[168]	Spillias	Australia	2024	GPT‐3.5‐Turbo, GPT‐4‐Turbo	Community‐based fisheries management (CBFM)
[169]	Tufanaru	Australia	2024	RobotSearch	Urinary tract infection (UTI)
[45]	Rokhshad	USA	2025	ChatGPT‐4, Gemini	Artificial intelligence applications in pediatric dentistry
[56]	Moens	Belgium	2025	ASreview, SWIFT Active Screener	Treatment efficacy in anesthesiology To investigate perturbations in gut microbiota Interventions to improve work participation in patients with chronic spinal pain Employment or return to work after implantable electrical neurostimulation in patients with chronic pain
[64]	Ghossein	Canada	2025	ChatGPT‐3.5, ChatGPT‐4, Google Bard, Meta Llama 2, Claude AI 2	Trauma hemorrhage
[72]	Janoudi	Canada	2025	Loon Lens	Evidence on the following drugs: Darolutamide (Nubeqa), Crisantaspase Recombinant (Rylaze), Upadacitinib (Rinvoq), Guselkumab (Tremfya), Lumasiran (Oxlumo), Mepolizumab (Nucala), Durvalumab (Imfinzi), and Finerenone (Kerendia).
[79]	Bayani	Canada	2025	Llama 3.2: 1b, Llama 3.2: 3b, Mistral 7B, Vicuna‐13B	Intersection between AI applications and the inclusion of people with disabilities throughout the process of AI application development
[87]	Lee	USA	2025	GPT‐4, GPT‐4o	Non‐small cell lung cancer, perinatal mood and anxiety disorders
[170]	Joos	Germany	2026	Llama‐3, Gemini 1.5 Flash, Claude 3.5 Sonnet, GPT‐4o, Llama 3.1, DeepSeek R1 0528, Qwen3, GPT OSS 20B, Llama 4 Scout, Llama 3.3, Claude Sonnet 4.5, Gemini 2.5 Flash, GPT‐5, GPT‐5 Mini, GPT‐5 Nano	Visual Network Analysis in Immersive Environments
[89]	Canfield	USA	2025	ASReview	Assessing overlapping therapies and potential cardiotoxicity within the Prostate Cancer disease space
[28]	Dai	China	2025	ChatGPT‐4o, Claude‐3.5 sonnet, Gemini‐1.5 pro, ASReview, Abstrackr	Comparison between sublobar resection and lobectomy in thoracic surgery
[29]	Lootus	USA	2025	AutoSLR	NR
[30]	Yao	Canada	2025	Covidence	Breast cancer clinical practice guide
[31]	Scherbakov	USA	2025	GPT‐4o, GPT‐4o mini	Use of NLP in mental health research
[32]	Thode	Sweden	2025	GPT‐3.5 Turbo, GPT‐4 Turbo, Llama 2 70B, Llama 2 7B, Mixtral 8×7B	Software quality measurement, modern code reviews
[33]	König	Germany	2025	Logistic Regression (LR), doc2vec, SBERT, TFIDF, Naive bayes, nn2layer, Random forest, Support vector machine (SVM)	Digital Technology Use and Adolescent Mental Health. Conflicts of Interest in Autism Research. Parental Mental Health and Child Behavior. Social‐Emotional Learning and Academic Outcomes. Nutritional Interventions and Cognitive Development. Childhood Obesity Prevention. Teacher Development and Student Outcomes. Reading Interventions. Exercise and Cognitive Function in Aging. Peer Mentoring and Academic Success. Financial Incentives in Mental Health Treatments. Sleep Interventions and Academic Performance. Stigma Towards Mental Illness. Emotional Intelligence Training in the Workplace. Ironic Effects of Social Integration. Loneliness Interventions for the Elderly. Mindfulness Interventions and Sleep Quality. Parental Involvement and Academic Achievement. Parental Influence on Infant Crying Gratitude Interventions and Psychological Health. Virtual Reality for Anxiety Treatment
[34]	Dogra	USA	2025	GPT‐4o, GPT‐4o mini, Gemini 1·5 pro, Gemini 2·0 flash, Llama 3·3	Functional connectivity changes following mild traumatic brain injury, as assessed by resting‐state functional MRI Performance of deep‐ learning models for creating synthetic post‐contrast T1‐ weighted MRI images from pre‐contrast MRI sequence inputs
[36]	López‐Pineda	Spain	2025	GPT‐4o mini, Llama 3 70B	Risk factors specific to postmenopausal women associated with the incidence of cardiovascular morbidity and mortality
[37]	Qin	China	2025	LightGBM (Light Gradient Boosting Machine)	CREAT random RCT dataset: RCT title‐and‐abstract citations of created between January 1, 2012, and January 1, 2023 CREAT cancer RCT dataset:randomly selected 5000 citations with “cancer” in the title or abstract
[38]	Chan	Australia	2025	BioBERT	Large dataset of systematic reviews from the Cochrane Library from 53 topics of Cochrane Review Group Code
[39]	Sciurti	Italy	2025	GPT‐4o mini, Llama 3.1 8B, Gemma 2 9B	Association between vaccine literacy and vaccination intention/status Impact of antibiotic exposure on antibioticresistant Acinetobacter baumannii isolation Efficacy of vitamin supplements in managing and preventing COVID‐19
[40]	Doneva	Switzerland	2025	GPT‐3.5 Turbo, GPT‐4‐turbo‐preview, Bio_ClinicalBERT, BERT‐base, BioBERT, PubMedBERT, BiomedBERT, BioLinkBERT, SciBERT	Neuroscience
[41]	Spiero	The Netherlands	2025	TF‐IDF, sBERT, Naive bayes, Logistic Regression (LR), Support vector machine (SVM)	Educational interventions for improving primary caregiver complementary feeding practices for children aged 24 months and under First‐line drugs inhibiting the renin angiotensin system versus other first‐line antihypertensive drug classes for hypertension Psychological therapies for treatment‐resistant depression in adults Anticoagulation for people with cancer and central venous catheters Face‐to‐face interventions for informing or educating parents about early childhood vaccination Antivascular endothelial growth factor for neovascular age‐related macular degeneration Interventions for implementation of thromboprophylaxis in hospitalized patients at risk for venous thromboembolism Completeness of reporting of clinical prediction models developed using supervised machine learning: A systematic review Performance of the Framingham risk models and pooled cohort equations for predicting 10‐year risk of cardiovascular disease: A systematic review and meta‐analysis Poor reporting of multivariable prediction model studies: Toward a targeted implementation strategy of the TRIPOD statement Prognostic models for mortality risk in patients requiring ECMO Prognostic models for radiation‐induced complications after radiotherapy in head and neck cancer patients The comparative and added prognostic value of biomarkers to the Revised Cardiac Risk Index for preoperative prediction of major adverse cardiac events and all‐cause mortality in patients who undergo noncardiac surgery
[43]	Li	USA	2025	GPT‐4	Relapsed and refractory multiple myeloma advanced melanoma
[44]	Scherbakov	USA	2025	GPT‐4o	Emergence of large language models as tools in literature reviews
[46]	Trad	Lebanon	2025	GPT‐4, Rayyan	Umbrella review on Vitamin D and Falls
[49]	Wong	USA	2025	SciBERT	Treatment outcomes related to distal radial fractures in older adults
[50]	Oami	Japan	2025	GPT‐4 Turbo, GPT‐3.5 Turbo	5 clinical questions (CQs) developed for the Japanese Clinical Practice Guidelines for Management of Sepsis and Septic Shock 2024
[51]	Wang	USA	2025	LEADS, GPT‐4o, GPT‐3.5, Haiku, Mistral‐7B, Dense	Simulation 1: Health, Musculoskeletal, Circulatory, Neoplasms, Mental, Infectious, Metabolic, Digestive, Nervous, Respiratory. Simulation 2: neurology, internal medicine, opthalmology, respiratory, dermatology, radiology, nephrology, gastroenterology
[52]	Kim	Korea	2025	GPT‐4o, GPT‐4o mini, Llama 3.1:8B	Cochrane drug intervention reviews
[53]	Insuk	Thailand	2025	ChatGPT‐4o, Claude 3.5 Sonnet	Pharmacological interventions for smoking cessation during pregnancy
[54]	Nitturi	USA	2025	Gemini Pro, ChatGPT‐ 4o‐mini	CNS Systematic Review and Evidence‐Based Guidelines for Patients With Chiari Malformation: Diagnosis
[55]	Bernard	France	2025	Elicit	Effectiveness of smart living environments in supporting ageing in place
[57]	Rokhshad	USA	2025	ChatGPT‐4, Claude 2 100k, Claude Instant 100k, Meta's LLaMA 3, Gemini	Tooth segmentation on dental radiographs using artificial intelligence
[58]	Cai	The Netherlands	2025	GPT‐4, GPT‐ 3.5, DeepSeek R1 Distill, Qwen2.5 7B, phi‐ 4 14B, Llama 3.1 8B, Gemma 2 27B, Claude2‐alpaca‐ 13B	Inflammatory bowel diseases (IBD), diabetes mellitus, sarcopenia, Glioma
[90]	Zhan	USA	2026	Review Copilot	Safety of using enteral formula with dietary fiber in hospitalized critical care patients associations between 100% orange juice and biomarkers of inflammation and oxidation in generally healthy populations interventions for reversing prediabetes in adults. Incidence of outcomes of patients with cancer who transitioned from one cancer drug to another versus patients who did not transition drugs
[171]	Koh	Australia	2026	ASReview	Empirical studies that administered mental health instruments in (1) the general population, (2) digital format, and (3) longitudinal designs
[60]	Oami	Japan	2025	GPT‐4o, Gemini 1.5 Pro, Claude 3.5 Sonnet, Llama 3.3 70B	Management of sepsis and septic shock in Japanese healthcare settings
[61]	Cao	Canada	2025	GPT‐4, GPT‐3.5 Turbo, GPT‐4 Turbo, Mixtral‐8×22‐0424, Mistral‐Large‐0224, Claude‐3.5‐Sonnet, Gemini Pro	Infectious diseases, Endocrinology and metabolism, Neurosciences and neurology, Pediatrics cardiovascular system and cardiology, Pediatrics respiratory system, Sepsis, Pharmacology and pharmacy, Cardiovascular system and cardiology, Endocrinology and metabolism surgery
[62]	Holland	Australia	2025	Research Screener	Non‐specific effects of respiratory vaccines for acute lower respiratory infections (ALRI) hospitalizations and related outcomes in children under 5 years of age
[63]	Liu	China	2025	ASReview	Application of deep learning in traumatic brain injury research
[91]	Kelley	USA	2026	GPT‐4	Core components of healthy marriage and relationship education (HMRE) programs that drive improvements in outcomes
[67]	Giannakis	USA	2025	ASReview	Impact of neuraxial anesthesia on perioperative hip fracture outcomes
[68]	Nykvist	Sweden	2025	GPT‐3.5‐0311, GPT‐3.5‐0613, GPT‐4‐1106	Electrification technologies for vehicles
[69]	Wu	USA	2025	ASReview	Development of a compu‐tational tool to improve food safety
[109]	Vivekanantha	Canada	2026	GPT‐5	Sports medicine, trauma and arthroplasty
[70]	Cassell	USA	2025	ISLaR 2.0	Cost‐effectiveness of adult pneumococcal vaccination
[71]	Zuo	USA	2025	ChatGPT‐3.5 Turbo	Relationship between stream fecal coliform concentrations and land use and land cover
[73]	Vembye	Denmark	2025	GPT‐3.5 Turbo, GPT‐4, GPT‐4o mini	Effects of functional family therapy (FFT) on drug abuse reduction for young people in treatment for nonopioid drugs effects of the FRIENDS preventive program on anxiety symptoms in children and adolescents conducted effects of testing frequencies on students' academic achievement
[92]	Parmar	USA	2026	GPT‐4 turbo, Claude‐3‐Sonnet, Gemini‐Pro‐1.0	Prostate cancer, renal cell carcinoma, hepatocellular carnicoma
[74]	Ito	Japan	2025	GPT‐4 turbo, GPT‐4o, Open AI o1	Nonpharmacological interventions for delirium in patients with cancer
[93]	Xu	USA	2026	ASReview, ChatGPT‐4, ChatGPT‐4o‐Mini	Learning analytics
[172]	Nordmann	Germany	2026	ChatGPT‐4, ChatGPT‐3.5, Rayyan	Digitally supported interprofessional communication and collaboration in healthcare
[75]	Al‐Marridi	Qatar	2025	GPT‐3.5 Turbo	Speech and language disorders
[76]	Boesen	Switzerland	2025	ASReview	Interventional trials of tumor‐infiltrating lymphocytes as the treatment for any type of cancer
[77]	Lin	USA	2025	GPT‐4o mini	Patient preferences for dementia interventions
[78]	Chen	China	2025	GPT‐3.5 Turbo, BioBERT	Precision oncology randomized controlled trials
[80]	Akinseloyin	UK	2025	GPT‐4o mini, Gemini 1.5 Flash, Claude 3 Haiku	Positron emission tomography (PET) and magnetic resonance imaging (MRI) for assessing tumor resectability in advanced epithelial ovarian/fallopian tube/primary peritoneal cancer Point‐of‐care ultrasonography for diagnosing thoracoabdominal injuries in patients with blunt trauma Transabdominal ultrasound and endoscopic ultrasound for diagnosis of gallbladder polyps Airway physical examination tests for detection of difficult airway management in apparently normal adult patients Xpert MTB/RIF assay for extrapulmonary tuberculosis and rifampicin resistance Noninvasive diagnostic tests for Helicobacter pylori infection Triage tools for detecting cervical spine injury in pediatric trauma patients Diagnostic tests for autism spectrum disorder (ASD) in preschool children Lower vs. higher oxygen concentrations titrated to target oxygen saturations during resuscitation of preterm infants at birth Nonpharmacological interventions for treating chronic prostatitis/chronic pelvic pain syndrome Antistreptococcal interventions for guttate and chronic plaque psoriasis Implantable miniature telescope (IMT) for vision loss due to end‐stage age‐related macular degeneration Melatonin for the promotion of sleep in adults in the intensive care unit Continuous intravenous perioperative lidocaine infusion for postoperative pain and recovery in adults Prescribed hypocaloric nutrition support for critically‐ill adults Educational interventions for improving primary caregiver complementary feeding practices for children aged 24 months and under Blue‐light filtering intraocular lenses (IOLs) for protecting macular health Subfascial endoscopic perforator surgery (SEPS) for treating venous leg ulcers Face‐to‐face interventions for informing or educating parents about early childhood vaccination Prophylactic vaccination against human papillomaviruses to prevent cervical cancer and its precursors Vaccines for preventing typhoid fever Antidepressants for insomnia in adults Anticoagulation for people with cancer and central venous catheters Psychological therapies for treatment‐resistant depression in adults Inhaled corticosteroids for bronchiectasis Methylphenidate for attention deficit hyperactivity disorder (ADHD) in children and adolescents, assessment of adverse events in nonrandomized studies Interventions for preventing occupational irritant hand dermatitis Comparison of a therapeutic‐only versus prophylactic platelet transfusion policy for people with congenital or acquired bone marrow failure disorders
[173]	Akinseloyin	UK	2026	GPT‐4o mini, Gemini 1.5 Flash, Claude 3 Haiku, Gemini 1.5 Pro	Positron emission tomography (PET) and magnetic resonance imaging (MRI) for assessing tumor resectability in advanced epithelial ovarian/fallopian tube/primary peritoneal cancer Point‐of‐care ultrasonography for diagnosing thoracoabdominal injuries in patients with blunt trauma Transabdominal ultrasound and endoscopic ultrasound for diagnosis of gallbladder polyps Airway physical examination tests for detection of difficult airway management in apparently normal adult patients Xpert MTB/RIF assay for extrapulmonary tuberculosis and rifampicin resistance Non‐invasive diagnostic tests for Helicobacter pylori infection Triage tools for detecting cervical spine injury in pediatric trauma patients Diagnostic tests for autism spectrum disorder (ASD) in preschool children Lower versus higher oxygen concentrations titrated to target oxygen saturations during resuscitation of preterm infants at birth Nonpharmacological interventions for treating chronic prostatitis/chronic pelvic pain syndrome Antistreptococcal interventions for guttate and chronic plaque psoriasis Implantable miniature telescope (IMT) for vision loss due to end‐stage age‐related macular degeneration Melatonin for the promotion of sleep in adults in the intensive care unit Continuous intravenous perioperative lidocaine infusion for postoperative pain and recovery in adults Prescribed hypocaloric nutrition support for critically ill adults Educational interventions for improving primary caregiver complementary feeding practices for children aged 24 months and under Blue‐light filtering intraocular lenses (IOLs) for protecting macular health Subfascial endoscopic perforator surgery (SEPS) for treating venous leg ulcers Face‐to‐face interventions for informing or educating parents about early childhood vaccination Prophylactic vaccination against human papillomaviruses to prevent cervical cancer and its precursors Vaccines for preventing typhoid fever Antidepressants for insomnia in adults Anticoagulation for people with cancer and central venous catheters Psychological therapies for treatment‐resistant depression in adults Inhaled corticosteroids for bronchiectasis Methylphenidate for attention deficit hyperactivity disorder (ADHD) in children and adolescents âĂŞ assessment of adverse events in nonrandomized studies Interventions for preventing occupational irritant hand dermatitis Comparison of a therapeutic‐only versus prophylactic platelet transfusion policy for people with congenital or acquired bone marrow failure disorders
[81]	Homiar	UK	2025	GPT‐4o	Prodopaminergic interventions for anhedonia
[82]	Teperikidis	USA	2025	Synthesa AI,	STEMI and Multivessel Percutaneous Coronary Intervention (PCI), Inhaled Reliever Therapies for Asthma, Antidepressants for Irritable Bowel Syndrome (IBS), Antiplatelet versus Anticoagulation Therapy in Heart Failure with Sinus Rhythm, APOC3 Antisense Oligonucleotides in Hypertriglyceridemia, Dexmedetomidine and Postoperative Delirium in Cardiac Surgery, Coadministration of Pneumococcal Vaccines with Influenza or SARS‐CoV‐2 Vaccines, Nebulized Antibiotics for Prevention of Ventilator‐Associated Pneumonia (VAP), Vitamin D Supplementation for Prevention of Acute Respiratory Infections
[83]	Sanghera	UK	2025	GPT‐3.5 Turbo, GPT‐4 Turbo, GPT‐4o, Llama 3 70B, Claude Sonnet 3.5, Gemini 1.5 Pro	Diabetes, Palliative Care, Carotid stenosis, Fertilization, Neonatology, Ophtalmology, Language learning, Dementia, Hyperbaric oxygen therapy, Stroke, Adult liver resection, Liver transplant, Chronic kidney disease, COVID‐19, Kidney stones, Acute otitis media in children, Dermatology, Neurology
[84]	Addison	USA	2025	Random forest, Logistic Regression (LR), Support vector machine (SVM)	Effects of parental substance use on children, experimentally induced masculinity threats
[85]	Fuller‐Tyszkiewicz	Australia	2025	LitQuest	Predictors, natural history and consequences of early patterns of relational health within family systems
[86]	Tao	China	2025	GPT‐4o, Kimi, Deep‐seeker	The safety assessment of RSV vaccines
[88]	Cawley	USA	2025	DoCTER	Two annual review series related to the MUP generally and the MUP in ambulatory care (ACMUP)
[174]	Uthman	UK	2022	Parallel convolutional neural network, Stacked convolutional neural network, Parallel stacked convolutional neural network, Recurrent neural network, Convolutional neural network ‐ Recurrent neural network	The primary prevention of CVD
[66]	Thurnham	USA	2024	Nested Knowledge Robot Screener	Clinical review informing evidence repository, clinical burden review, clinical review mental health review, clinical review informing virtual patient creation, clinical review ‐ adverse events, economic review
[95]	Cichewicz	USA	2024	Nested Knowledge Robot Screener	Clinical efficacy and safety economic burden and evaluation humanistic burden and utilities
[175]	Felizardo	Brazil	2024	ChatGPT‐4	Convergence of Human‐Computer Interaction and Artificial Intelligence User profiles in games or gamified environments and evaluate the impact of game elements within these environments based on the users' profiles
[176]	Schopow	Germany	2023	ChatGPT‐3.5 legacy	Applications of the Natural Language Processing Tool ChatGPT in Clinical Practice
[110]	Gates	Canada	2020	Abstrackr	Biomarkers, Brain injury, Activity and pregnancy, Concussion, Antipsychotics, Digital technologies for pain, Treatments for bronchiolitis, VBAC, Visual acuity, Experience of bronchiolitis, Experiences of UTIs, Preterm delivery, Community gardening, Depression safety, Depression treatments, Patient education for cancer, Workplace stress
[177]	Valizadeh	Iran	2022	Rayyan	Applied machine learning algorithms on cerebral structural magnetic resonance imaging (sMRI). Applied machine learning algorithms on cerebral resting‐state functional magnetic resonance imaging (rs‐fMRI). Applied machine learning algorithms on electroencephalogram (EEG)

b. Experiences regarding the use of automation AI resources				
References	First author's surname	Corresponding authors' stated country	Year	Type of article
[136]	van de Schoot	Netherlands	2020	Design, development, and implementation study
[113]	Wagner	Canada	2020	Essay
[178]	Feng	China	2022	Systematic literature review
[179]	Schmidt	UK	2022	Systematic literature review
[180]	Li	China	2022	Feature selection analysis
[181]	Cierco Jimenez	Spain	2022	Mapping review
[182]	Harrison	UK	2020	Systematic literature review
[183]	Khalil	Australia	2022	Scoping review
[103]	Cowie	USA	2022	Systematic literature review
[184]	Cleo	Australia	2019	Mixed method design
[185]	Wilson	UK	2023	Mixed method design
[104]	Hou	USA	2024	Narrative review
[186]	Halman	Australia	2024	Communication paper
[187]	Bannach‐Brown	Scotland	2019	Design, development and implementation study
[105]	Jap	USA	2019	Design, development and implementation study
[188]	van der Mierden	Germany	2019	Feature analysis
[189]	Chappell	UK	2023	Commentary
[96]	Adam	USA	2022	Design, development and implementation study
[190]	Marshall	UK	2019	Commentary
[191]	Westgate	Australia	2019	Commentary
[192]	van Dijk	Netherlands	2023	Communication paper
[163]	Khraisha	Ireland	2024	Design, development, and implementation study
[160]	Pilz	Germany	2024	Design, development, and implementation study
[193]	Sandner	Austria	2024	Survey paper
[19]	O'Connor	USA	2019	Commentary
[120]	Yao	Canada/China	2024	Systematic literature review
[18]	König	Germany	2024	Design, development and implementation study
[119]	Perlman ‐ Arrow	Canada	2022	Design, development, and implementation study
[45]	Rokhshad	USA	2025	Design, development, and implementation study
[56]	Moens	Belgium	2025	Design, development, and implementation study
[64]	Ghossein	Canada	2025	Feasibility study
[79]	Bayani	Canada	2025	Design, development, and implementation study
[170]	Joos	Germany	2026	Design, development and implementation study
[89]	Canfield	USA	2025	Design, development, and implementation study
[28]	Dai	China	2025	Diagnostic study
[32]	Thode	Sweden	2025	Experimental simulation
[33]	König	Germany	2025	Comparative analysis
[194]	Yanxi	China	2026	Systematic literature review
[34]	Dogra	USA	2025	Design, development, and implementation study
[36]	López‐Pineda	Spain	2025	Design, development, and implementation study
[38]	Chan	Australia	2025	Design, development, and implementation study
[39]	Sciurti	Italy	2025	Feasibility study
[41]	Spiero	Netherlands	2025	Design, development, and implementation study
[42]	Clark	Australia	2025	Systematic literature review
[44]	Scherbakov	USA	2025	Systematic literature review
[48]	Sen	USA	2025	Editorial
[53]	Insuk	Thailand	2025	Design, development and implementation study
[57]	Rokhshad	USA	2025	Design, development, and implementation study
[59]	Xu	China	2025	Systematic literature review
[60]	Oami	Japan	2025	Design, development, and implementation study
[65]	Gaelen	USA	2025	Rapid review
[66]	Kallmes	USA	2025	Methodological framework
[109]	Vivekanantha	Canada	2026	Design, development, and implementation study
[70]	Cassell	USA	2025	Case study
[73]	Vembye	Denmark	2025	Design, development, and implementation study
[92]	Parmar	USA	2026	Case study
[74]	Ito	Japan	2025	Design, development, and implementation study
[172]	Nordmann	Germany	2026	Feasibility study
[76]	Boesen	Switzerland	2025	Case study
[78]	Chen	China	2025	Design, development, and implementation study
[81]	Homiar	UK	2025	Design, development, and implementation study
[83]	Sanghera	UK	2025	Design, development and implementation study
[84]	Addison	USA	2025	Design, development, and implementation study
[85]	Fuller‐Tyszkiewicz	Australia	2025	Design, development, and implementation study
[175]	Felizardo	Brazil	2024	Design, development, and implementation study
[177]	Valizadeh	Iran	2022	Diagnostic study

### 3.3 Authors' Intended Purpose for Using AI Resources

**Figure 6:**
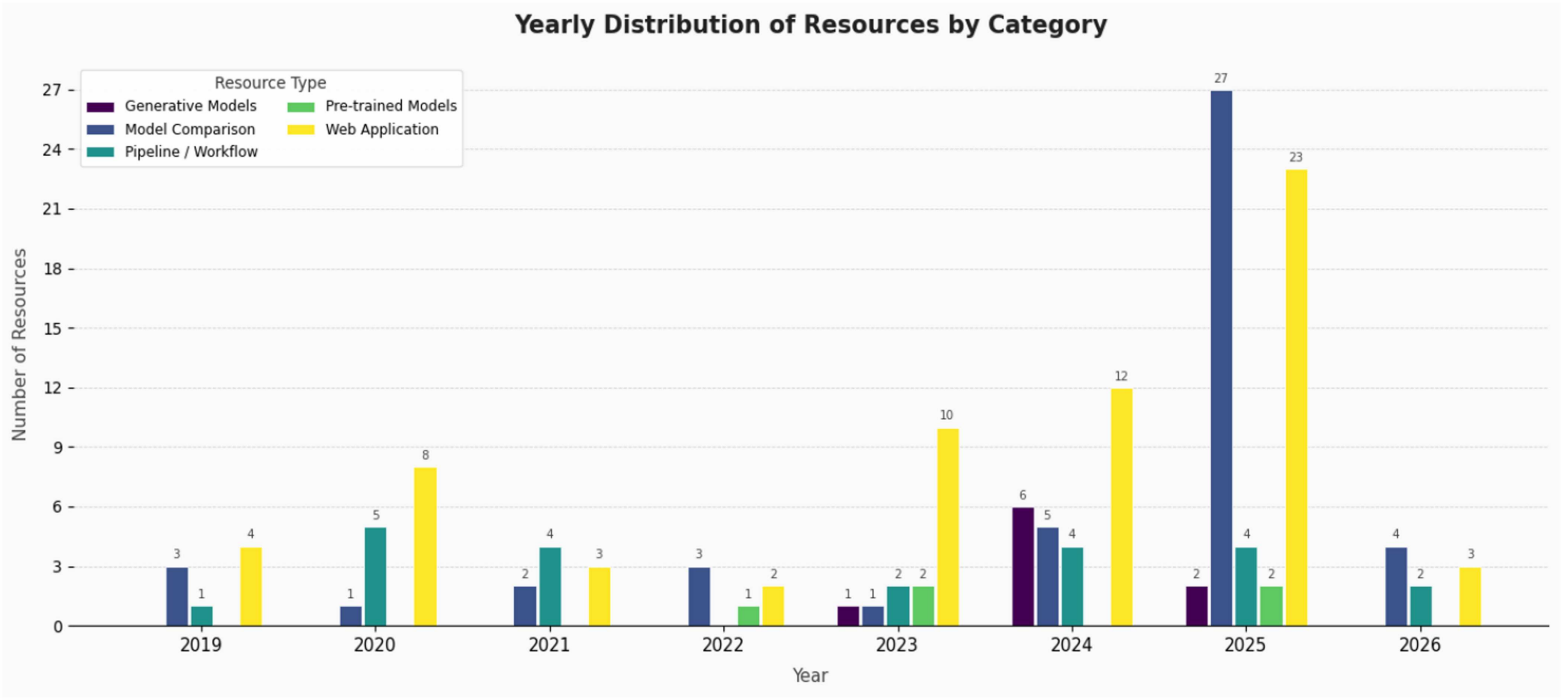
Bar graph regarding the frequency of AI resources used in the automation of screening studies for systematic literature reviews(*Source:* Authors' elaboration). Figure code generated using Claude 4.6 Sonnet and rendered via Google Colab (Python). Final layout edited and verified by the authors. Distribution of the different types of AI resources used for supporting automation during the screening phase of systematic literature reviews according to year of publication.

Web applications were the most reported resource (*n* = 62) [4, 9, 29, 30, 35, 45, 53, 54, 55, 56, 57, 63, 64, 67, 69, 70, 71, 72, 76, 82, 85, 88, 89, 93, 94, 95, 97, 99, 101, 106, 108, 110, 112, 114, 118, 119, 122, 123, 124, 128, 132, 136, 138, 139, 141, 148, 149, 150, 151, 152, 153, 158, 161, 163, 167, 169, 171, 172, 175, 176, 177] whilst pretrained models were the least reported (*n* = 7) [38, 49, 129, 131, 137, 140, 144] (Figure 6. Bar graph regarding the frequency of AI resources used in the automation of screening studies for systematic literature reviews). The most frequently described web application was ASReview during 2025 (*n* = 7) [28, 56, 63, 67, 69, 76, 89]; however, Abstrackr was the tool which was most consistently mentioned in the publications (*n* = 10) [9, 28, 97, 99, 110, 114, 118, 124, 128, 139] (Appendix S4a. Heatmap regarding type of AI resources by year—Web applications). Regarding articles grouped as comparative models, support vector machines (SVM) were reported most during the target years (*n* = 12) [107] (Appendix S4b. Heatmap regarding type of AI resources by year—Model comparison). Regarding generative models, GPT‐3.5 Turbo was the most described (*n* = 7) [47, 126, 130, 134, 142, 145, 146], such models being mostly reported during 2024 and 2025 (Appendix S4c. Heatmap regarding type of AI resources by year—Generative Models and Pretrained models). PubMedBERT was the most reported in the pretrained model category (*n* = 3) [129, 137, 144]. Most articles regarding the pipeline and workflow category were published during 2020 (*n* = 5) [8, 100, 102, 111, 159], workflows regarding different steps for random forest models being the most reported (*n* = 2) [115, 143] (Appendix S4d. Heatmap regarding type of AI resources by year—Pipeline/Workflow).

### 3.4 Comparing Automation Resources to Other Screening Methodologies

Unique retrospective comparison was the most common comparator used regarding AI resources, human reviewers using classical review techniques (*n* = 91) [8, 9, 16, 18, 29, 30, 32, 33, 34, 35, 36, 38, 39, 41, 43, 46, 47, 49, 50, 51, 52, 54, 55, 56, 61, 64, 67, 68, 69, 72, 73, 74, 77, 80, 81, 82, 83, 84, 85, 86, 87, 88, 89, 90, 97, 100, 101, 102, 106, 107, 110, 111, 112, 114, 115, 116, 117, 121, 123, 125, 127, 129, 131, 132, 133, 134, 135, 136, 138, 140, 142, 143, 144, 147, 151, 153, 154, 156, 157, 158, 159, 162, 165, 166, 167, 168, 170, 171, 172, 173, 175]. This comparator relied on an existing data set, either retrieved from established repositories or derived from databases previously assembled by the study authors. The least used being comparison of different stopping criteria for model calibration (*n* = 1) [161] (Appendix S5. Comparing automation resources to other screening methodologies). Four articles [99, 118, 152, 164] included two types of comparators: a concurrent comparison with human reviewers using the traditional review process, and a concurrent comparison with a single human reviewer. Six articles [9, 43, 58, 82, 132, 156] also used two comparators: a concurrent comparison with human reviewers using the traditional process, and a retrospective comparison with the same type of reviewers.

Criterion assessment was the category which most reported metrics (*n* = 134) [8, 9, 16, 18, 28, 29, 30, 31, 32, 33, 34, 35, 36, 37, 38, 39, 40, 41, 43, 44, 45, 46, 47, 49, 50, 51, 52, 53, 54, 55, 56, 57, 58, 60, 61, 63, 69, 70, 71, 74, 75, 77, 78, 81, 82, 83, 84, 85, 86, 87, 88, 90, 93, 94, 95, 97, 98, 99, 100, 101, 102, 106, 107, 108, 109, 110, 111, 112, 114, 115, 116, 117, 118, 119, 121, 122, 124, 125, 126, 127, 128, 129, 130, 131, 133, 134, 135, 137, 138, 139, 140, 142, 143, 144, 145, 146, 147, 148, 149, 150, 151, 152, 154, 155, 156, 157, 159, 160, 161, 162, 163, 164, 165, 166, 167, 168, 169, 170, 171, 172, 173, 174, 175, 176, 177], followed by utility (*n* = 88) [4, 8, 9, 16, 29, 30, 33, 37, 39, 41, 44, 46, 47, 49, 50, 51, 56, 58, 60, 61, 62, 67, 68, 69, 70, 71, 75, 76, 80, 81, 82, 83, 84, 85, 86, 88, 89, 90, 92, 93, 97, 99, 100, 101, 108, 109, 110, 111, 112, 114, 115, 116, 117, 119, 123, 124, 125, 126, 127, 128, 130, 131, 132, 135, 136, 138, 139, 141, 142, 143, 144, 145, 150, 153, 154, 155, 158, 159, 160, 161, 162, 164, 165, 167, 168, 169, 172, 173]. When results were examined by resource type, both type of assessments appeared in all categories, (Appendix S6. Bar graph regarding type of assessment by resource category).

#### 3.4.1 Criterion Assessment

**Figure 7:**
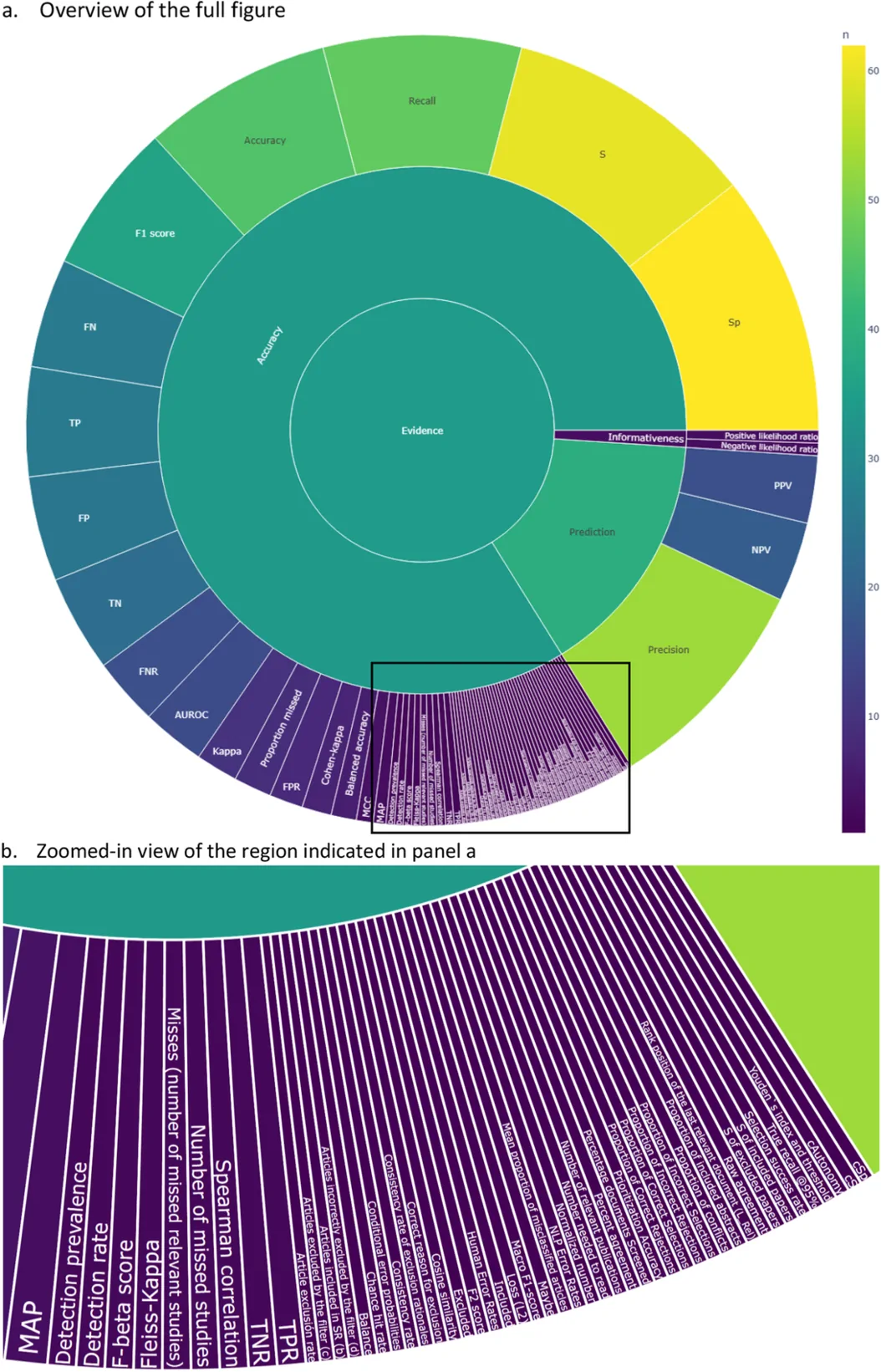
Sunburst diagram displaying reported metrics for evidence concerning criterion assessment (*Source:* Authors' elaboration). (a) Overview of the full figure. Figure code generated using ChatGPT 3.5 and rendered via Google Colab (Python). Final layout edited and verified by the authors. Sunburst diagram displaying the different types of metrics reported when performance evaluation was assessed. The inner rings represent the three main categories, while the outer rings display their corresponding metrics. Segment size indicates each category's relative frequency within the studies analyzed. (b) Zoomed‐in view of the region indicated in (a) Zoomed‐in view of a selected region from the sunburst diagram in panel a, providing a more detailed visualization of the corresponding metrics and their relative frequencies within the studies analyzed.

**Figure 8:**
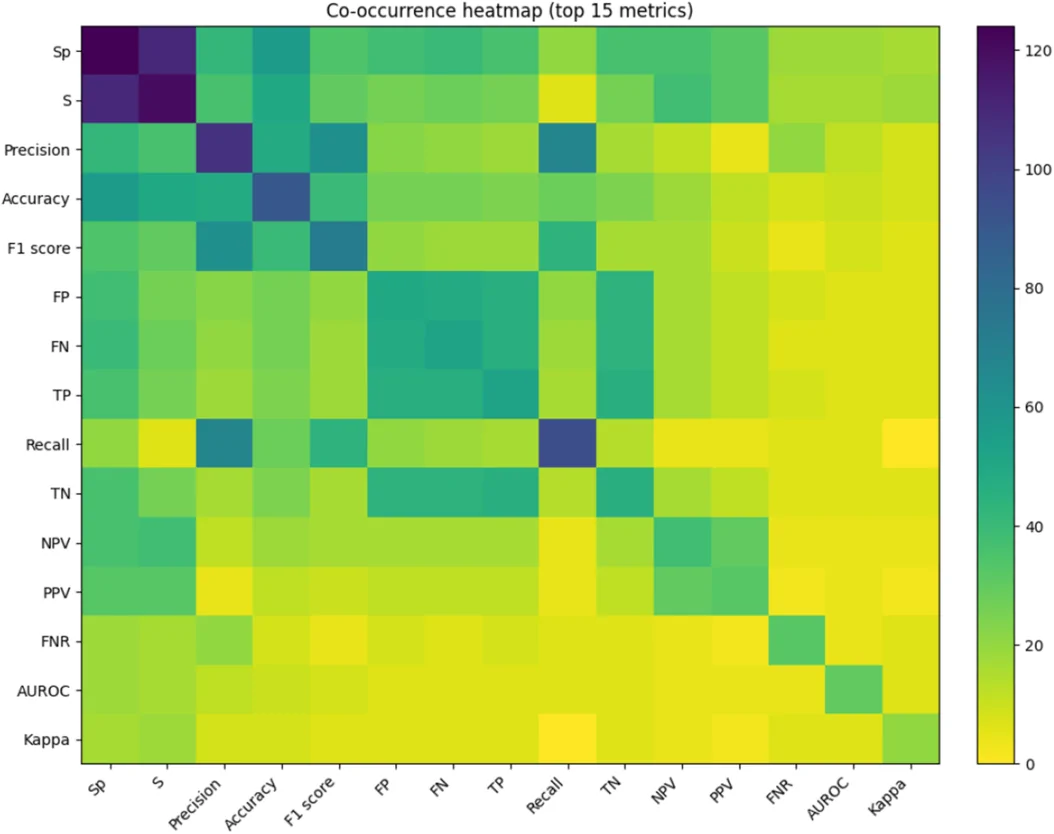
Co‐occurrence heatmap of the performance metrics most frequently reported in the studies included here (*Source:* Authors' elaboration). Figure code generated using ChatGPT 3.5 and rendered via Google Colab (Python). Final layout edited and verified by the authors. Co–occurrence frequency for the 15 most frequently reported performance metrics.

Accuracy accounted for the largest amount of reported metrics, specificity being the most frequently used (*n* = 61) [16, 18, 28, 31, 34, 35, 36, 37, 39, 43, 44, 45, 50, 54, 57, 60, 61, 63, 64, 68, 70, 71, 72, 73, 74, 77, 81, 82, 83, 87, 90, 93, 97, 106, 109, 111, 115, 118, 122, 124, 125, 126, 127, 128, 130, 131, 133, 134, 139, 140, 142, 147, 149, 151, 152, 156, 161, 163, 172, 176, 177]. The second group had the least metrics reported: positive (*n* = 3) [36, 122, 161] and negative likelihood ratio (*n* = 3) [36, 122, 161]. Similarly, the third group of metrics included a few metrics, precision (*n* = 52) [16, 29, 31, 32, 38, 40, 41, 43, 44, 45, 49, 52, 53, 56, 57, 58, 63, 68, 70, 72, 75, 78, 79, 83, 84, 86, 87, 92, 94, 95, 102, 107, 111, 115, 117, 119, 124, 128, 129, 135, 137, 139, 144, 155, 156, 157, 160, 165, 166, 172, 174, 176] being the most reported (Figure 7. Sunburst diagram displaying reported metrics for evidence concerning criterion assessment). Figure 8 gives a co‐occurrence heatmap of the most frequently reported performance metrics in the included studies. Darker shades indicate higher frequencies of co‐occurrence between pairs of metrics. The main diagonal (from top left to bottom right) represents the total number of studies that reported each metric. Off‐diagonal intersections represent common combinations of metrics. For instance, a dark cell between “Sensitivity” (row) and “Specificity” (column) indicates that many studies reported both metrics together. Lighter areas (lower values) show metric pairs that were rarely reported in combination. Co‐occurrence heatmap revealed frequent joint reporting of classical diagnostic indicators such as sensitivity, specificity and precision. By contrast, metrics commonly used in ML, such as AUROC, F1‐score, and precision, had fewer and less consistent co‐occurrences.

Authors' definitions and formulas for exploring trends, similarities and/or differences are reported (Appendix S6. How the metrics reported regarding Criterion assessment have been defined in the relevant papers selected here).

#### 3.4.2 Utility Assessment

**Figure 9:**
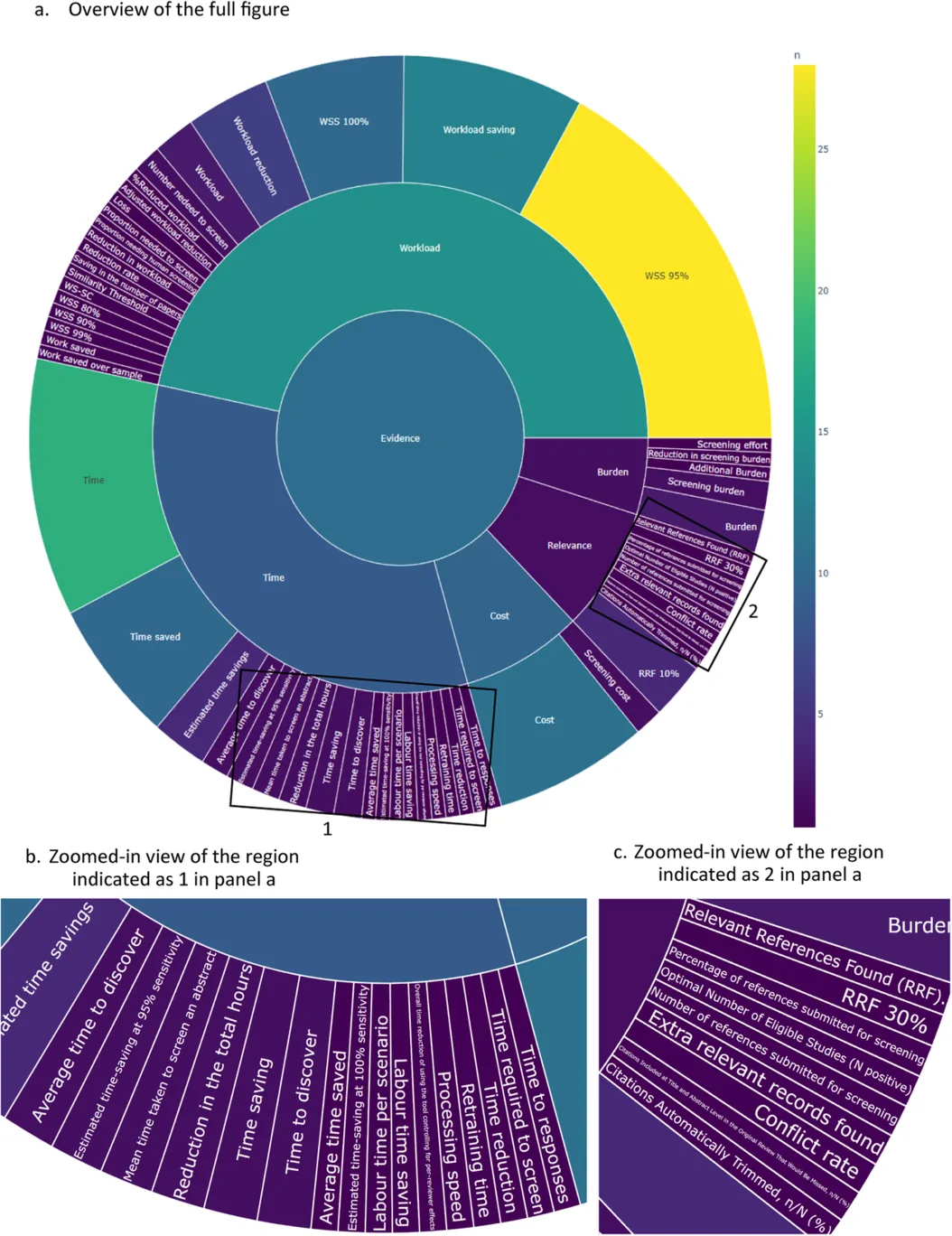
Sunburst diagram displaying reported metrics for evidence concerning utility assessment.(*Source:* Authors' elaboration). (a) Overview of the full figure. Figure code generated using ChatGPT 3.5 and rendered via Google Colab (Python). Final layout edited and verified by the authors. Sunburst diagram displaying the different types of metrics reported regarding utility evaluation. The inner rings represent the five main categories, while the outer rings display their corresponding metrics. Segment size indicates each category's relative frequency within the studies analyzed here. (b) Zoomed‐in view of the region indicated as 1 in (a). Zoomed‐in view of a selected region from the sunburst diagram in panel a, providing a more detailed visualization of the corresponding metrics and their relative frequencies within the studies analyzed. (c) Zoomed‐in view of the region indicated as 2 in (a). Zoomed‐in view of a selected region from the sunburst diagram in panel a, providing a more detailed visualization of the corresponding metrics and their relative frequencies within the studies analyzed.

Most articles focused on workload, where the most often‐mentioned metric was work saved over sampling (WSS) at no less than 95% recall (*n* = 28) [30, 41, 62, 68, 69, 70, 80, 85, 89, 93, 100, 101, 117, 123, 125, 132, 135, 136, 138, 143, 153, 154, 158, 160, 162, 165, 168, 173], followed by workload saving (*n* = 12) [9, 39, 110, 114, 115, 124, 126, 128, 131, 139, 161, 172]. Different approaches to measurement were reported in the time category, time measurement being the most frequently used metric (*n* = 18) [4, 47, 49, 50, 51, 60, 61, 71, 75, 80, 84, 86, 108, 109, 145, 153, 154, 164], followed by time saved (*n* = 10) [41, 44, 88, 90, 97, 112, 114, 115, 116, 124]. Regarding relevance metrics, screening 10% of the total number of records was the most frequently identified relevant reference (*n* = 4) [33, 136, 158, 173]. Furthermore, cost was reported (*n* = 11) [39, 47, 60, 61, 71, 75, 80, 101, 109, 154, 172] along with burden in the articles (*n* = 3) [99, 133, 138] (Figure 9. Sunburst diagram displaying reported metrics for evidence concerning utility assessment).

The definitions and ways of operationalising reported metrics varied widely; authors often used the same terms for describing different concepts and applied distinct formulas or denominators to express similar outcomes (Table 2).

**Table 2:** How the metrics reported regarding utility assessment have been defined in the relevant papers selected here.

Category	Metric	Description
Burden	Burden	− Reduce the number of publications to be examined manually [133] − Proportion of citations screened to identify all included studies [99] − Percentage reduction of articles to screen with the use of a machine learning–assisted platform [138]
	Reduction in screening burden	− The number of records not required to be screened [112]
	Screening burden	− The complete sample of studies needed to be screened to include all relevant records [141] − Percentage decrease in the number of abstracts requiring human review, relative to full manual screening [82]
	Screening effort	− The number of records that must be screened to achieve a specified 95% recall [171]
	Additional Burden	− The work saved when the criterion was triggered subtracted from the work saved when the recall target was actually achieved [159]
Cost	Cost	− Reflects the extra percentage of documents screened in order to obtain a given level of estimated recall. Cost=PS_Obtained−PS_Theory [101] − Economic resources required for the article screening process, expressed as the amount of money saved (USD) [39, 47, 60, 61, 71, 75, 80, 109, 154, 172]
	Screening cost	− The proportion of articles screened until the stopping rule criteria were met [18] − Ratio of abstracts that have been screened 1−WSS [33]
Relevance	citations Automatically Trimmed, n/N (%)	− To trim the number of citations to be manually screened by humans automatically. This is based on the output of GPT models using two different rules of combination for reporting the PICOS element for a given citation [142].
	Optimal number of eligible studies (N positive)	− The %Reduced workload was estimated by fixing the recall rate at 100% (i.e., sensitivity), plotted against the number of eligible studies used for training (N positive) for each scenario. The optimal N positive was estimated using the Kneedle algorithm, which identifies the knee point of the graph [155]
	Relevant references found (RRF).	− The RRF value indicates the proportion of relevant citations that are found after screening a portion of all citations [165]
	RRF%10	− The proportion of the total number of relevant records found at screening 10% of the total number of records available in the data set [33, 136, 158, 173]
	RRF 30%	− Relevant records found after screening the first 30% of the total records [138]
	Number of references submitted for screening	− Number of references from the original data set that were screened by the AI resource before meeting the screening criterion [76]
	Percentage of references submitted for screening	− Percentage of references from the original data set that were screened by the AI resource before meeting the screening criterion [76]
	Citations included at title and abstract level in the original review that would be missed n/N (%)	− Citations included at title and abstract level in the original review that would be missed with the use of GPT [142]
	Extra relevant records found	− Additional proportion of relevant records found at a given proportion of total records screened compared to random screening [69]
	Conflict rate	− Proportion of abstracts requiring conflict resolving votes [119]
Time	Estimated time‐saving at 95% sensitivity	− How much time can be saved after finding 95% of the relevant studies for screening [30, 127]
	Estimated time‐saving at 100% sensitivity	− How much time can be saved after finding 100% of the relevant studies for screening [30]
	Mean time taken to screen an abstract	− Two‐sided unequal variances *t*‐tests to compare mean time taken per abstract with tool versus without‐tool for the three sets of abstracts described [119] − Time in seconds taken to screen a single abstract [70]
	Reduction in the total hours	− Reduction in hours to perform the screening process [46, 169]
	Time	− Time to perform the screening [4, 47, 49, 50, 51, 60, 61, 71, 75, 80, 84, 86, 109, 145, 153, 154] − Reduction in hours of reading time [164] − The count of irrelevant records since the last selection of a relevant one [108]
	Time reduction	− Reduction in hours of work [111]
	Time required to screen	− The mean time taken to complete the review [118]
	Time saved	− Mean title/abstract screening hours saved [44, 88, 112] − The estimated time saved by not screening records manually. We assumed a screening rate of 0.5 min per record and an 8‐h workday [114] − Reduction in person hours associated with the workload reduction [90, 115] − Time saved through automation was calculated by multiplying the median screening time per reference by the number of references automatically screened [116] − Hours saved in screening, an average duration of 30 s was considered as the time needed to screen one record [41] − Time saved based on the records that would not need to be screened; estimated based on a screening rate of 0.5 min per record. Time savings = #records predicted as irrelevant (true negatives + false negatives) x (0.5 min/record) [97, 124]
	Time saving	− Time that could be saved by using a specific stopping rule could be calculated by multiplying the PNS by the time required for screening one title and abstract (45 s) [150] − Time saving* = [(titles predicted as irrelevant × 0.5)/60]/8 [139]
	Average time saved	− Average reduction in person hours during screening [126]
	Estimated time savings	− Reduction in time required for screening [9, 29, 110]
	Overall time reduction	− Direct indicator of whether screening time was reduced with the tool. Modeled time taken as a function of a tool‐usage using a Gaussian GLM with an identity link function [119]
	Processing speed	− Time taken to complete screening process [130]
	Retraining time	− Retraining time required in seconds to reflect computational intensity [117]
	Time to discover	− The number of records that must be screened before a specific relevant paper is detected [69, 173]
	Labor time per scenario	− Sum of the time spent by all human reviewers on screening the data set under the scenario [37]
	Labor time saving	− One minus the ratio of the new labor time to the labor time of the standard manual screening, between 0 and 1. A higher labor time saving indicates a better practical performance of the ML [37].
	Time to responses	− Overall time needed to screen records [39]
	Average time to discover	− The Average time to discovery (ATD) is then given by taking the average of all average‐record‐TD's and expressed as a fraction of the total number of records needed to screen [69, 173]
Workload	Proportion needed to screen	− Proportion of the dataset that needs to be screened to achieve complete recall [150]
	Reduction in workload	− Decrease in the amount of manual screening required when an automated tool [144]
	Reduction rate	− The percentage of articles excluded by the indicated rapid medical evidence synthesis (RMES) filter, calculated as the number of articles excluded divided by the number of articles assessed. A value of 0% indicates that the RMES filter retained all of the PubMed‐cited articles with an abstract that were used by the authors in their SR. A value of 100% indicates that the RMES filter incorrectly excluded all those articles [167].
	Workload	− Proportion of citations that a human reviewer will have to manually review. # of citations predicted as relevant (true positives + false positives)/total number of records [97] − Documenting the total duration of the citation screening session [149] − Reduction in screened articles [67]
	Workload reduction	− Percentage reduction in the number of articles that reviewers must evaluate from the articles identified as potentially relevant at the end of the screening process [8] $\mathrm{WLR}=\frac{\mathrm{PE}+0.5\times \mathrm{PI}}{N}.$ − *N* is the total number of title/abstracts, PE: predicted exclude, PI: predicted include, 0.5 is to account for one of the two reviewers who screen the PI studies [16] − $\frac{n_{\mathrm{records}\;\mathrm{excluded}\;\mathrm{by}\;\mathrm{model}}}{n_{\mathrm{all}\;\mathrm{records}}}$ [58] − Number of records not needed to screen (assuming the reviewer could stop screening at 95% recall) [41] − Number of articles that the model automatically excludes (and that the human no longer has to review), assuming that those without an abstract still require manual review [81] − Number of correctly excluded articles per 100 screened records [83]
	Workload saving	− The proportion of articles that would not need to be screened manually [9, 110, 114, 124, 139] − The difference between the total number of abstracts and the number of abstracts screened by the workflow, assuming each abstract is screened by two reviewers [115] − Subtracting the time required for human screening and the time required to use GPT models [126] − Workload saving (citations) The percentage of citations screened by reviewer 2 before predictions were generated by Abstrackr [128] − Workload saving (time) The percentage of time spent reviewing by reviewer 2 relative to reviewer 1 who screened all citations[128] − Ratio of the number of citations predicted to be ineligible to the total number of citations [131] − Proportion of citations that were correctly identified as irrelevant, thereby reducing the workload for human reviewers $\frac{\mathrm{TN}}{\mathrm{TN}+\mathrm{FN}+\mathrm{TP}+\mathrm{FP}}$ [39, 161, 172]
	Work saved	− $\frac{\mathrm{Total}\;\mathrm{samples}-(\mathrm{TP}+\mathrm{FP})}{\mathrm{Total}\;\mathrm{samples}}$ [92]
	Work saved over sample	− $\frac{\mathrm{Work}\;\mathrm{saved}}{\mathrm{Loss}}$ [92]
	Loss	− False exclusion of articles that were meant to be included [92]
	WSS 95%	− Reduction in publications needed to be screened, at a 95% level of recall related to the relevant citations. Yields an estimate of the amount of work that can be saved at the cost of failing to identify 5% of relevant citations [30, 41, 62, 68, 69, 70, 80, 85, 89, 100, 101, 117, 123, 125, 132, 135, 136, 138, 143, 153, 154, 158, 160, 162, 165, 168, 173, 195].
	WS‐SC	− Work saved when the stopping criteria were met [159]
	WSS 100%	− Reduction in publications needed to be screened, at a recall of 100%, all relevant literature on the topic is retrieved [30, 62, 69, 132, 136, 138, 154, 158, 162, 168]
	WSS 90%	− Reduction in publications needed to be screened, at a 90% level of recall related to the relevant citations [69]
	WSS 99%	− Reduction in publications needed to be screened, at a 99% level of recall related to the relevant citations [69]
	WSS 80%	− Reduction in publications needed to be screened, at a 80% level of recall related to the relevant citations [89]
	Adjusted workload reduction	− Adjustment made for the scenario of using one reviewer instead of two by automating part of the screening process [141]
	%Reduced workload	− $\frac{N-(\mathrm{TP}+\mathrm{FP})}{N}\times 100,$ where TP and FP are true and false positive studies (predicted eligible studies), and *N* is the total number of studies for each SR [155]
	Number needed to screen	− NNS=TP+FP where TP and FP are true and false positive studies [155] − Number of articles to screen/read to identify a relevant article [56]
	Similarity Threshold	− Similarity between studies was assessed using a cosine similarity threshold that represented the distance between 2 vector representations for each study within a paired sample $\mathrm{Cosine}\;\mathrm{similarity}\;\mathrm{score}=\;\frac{\vec{A}∙\vec{B}}{\left\|\vec{A}\right\|∙\left\|\vec{B}\right\|},$ where $\vec{A}$ and $\vec{B}$ are vector representations of the first and second studies within a paired sample. The cosine similarity score ranges from 1 to –1, where 1 represents perfect similarity between both studies and –1 represents complete dissimilarity enabling quantification of the degree of similarity between pairs of studies and the identification of potentially relevant studies based on their vector representations [155].
	Proportion needing human screening	− Number of papers screened to stop criteria divided by the total number of articles to be screened [85]
	Saving in the number of papers	− Mean percentage of papers that did not have to be reviewed [132]

*Source:* Authors' elaboration.

### 3.5 Authors' Reflections and Recommendations Concerning the Use of AI Resources During Screening

The commonest types of article were those dealing with the design, development and implementation of AI resources (*n* = 29) [18, 34, 36, 38, 41, 45, 53, 56, 57, 60, 73, 74, 78, 79, 81, 83, 84, 85, 89, 96, 105, 109, 119, 136, 157, 163, 164, 175, 187], followed by SLRs (*n* = 9) [42, 44, 59, 103, 120, 178, 179, 182, 194]. The main concerns were centered on the need for standardizing best practices, particularly regarding the persistence of the human component in the process (*n* = 26) [32, 36, 39, 42, 44, 45, 48, 53, 56, 57, 60, 66, 73, 74, 79, 81, 83, 84, 92, 103, 157, 164, 172, 175, 185, 192] and when and how to use the tools appropriately (*n* = 17) [32, 33, 34, 59, 73, 78, 81, 83, 84, 85, 109, 120, 163, 172, 175, 191, 192] (Table 3).

**Table 3:** Key considerations regarding the use of AI resources in automated screening.

Sensemaking and document classification	Consideration	References
Best practices regarding tool use	Appropriately used	[32, 33, 34, 59, 73, 78, 81, 83, 84, 85, 109, 120, 163, 172, 175, 191, 192]
	Persistence of the human component in the process	[32, 36, 39, 42, 44, 45, 48, 53, 56, 57, 60, 66, 73, 74, 79, 81, 83, 84, 92, 103, 157, 164, 172, 175, 185, 192]
	Transparency reporting choices made by the model	[48, 65, 66, 76, 83, 136, 157, 192, 194]
	Supporting living reviews	[103]
	Evaluating AI resources	[89]
	Combining classification algorithm model features for achieving the best outcome	[34, 39, 60, 157, 180, 194]
Fields for future research	Compatibility for recombination	[33, 53, 64, 81, 96, 113, 181]
	Transparency and replicability	[64, 113, 178, 183]
	Evaluation and validity	[28, 36, 53, 65, 70, 81, 113]
	Usability, training and guidelines	[53, 89, 113, 175]
	Proposing a metric for evaluating automated screening accuracy	[104]
Lack of standardization	Stopping criteria	[18, 41, 65, 96, 104, 187, 190, 192]
	Metrics in the evaluation of resources for automating SRL	[32, 73, 119, 164, 177, 178, 183, 189]
	Benchmark data sets for evaluating resources against a gold standard	[19, 53, 105, 179, 183]
	Study designs for evaluating resources for automating SLR	[19, 41, 57, 65, 120, 189, 194]
AI resource characteristics	Characteristics of resources facilitating usability (user interface, interoperability)	[36, 38, 109, 182, 184, 193, 194]
	Feature assessment	[186, 188]

*Source:* Authors' elaboration.

## 4 Discussion

This review has presented the available evidence regarding AI resources used for automating the screening of SLRs, having described the names of the metrics used for validating automation outcomes and summarized experiences regarding their use. It is evident that this is a rapidly growing and evolving field which is closely linked to technological advances. The majority of studies utilize a single data set, with median data sets sample sizes remaining consistent across different publication periods and data sets counts (based on exploratory descriptive analysis). Notably, these observed sample sizes align with established machine learning requirements: while training models from scratch typically necessitates thousands of records to ensure stability, validating existing state‐of‐the‐art AI with a dataset of approximately 800 yields statistically significant and reliable results [196]. However, important gaps remain, for example, the lack of standardizing phases or objectives arising from comparisons with any other screening methodology and the lack of consensus concerning what could be the best standards for making comparisons. Arising from comparison with any other screening methodology, this research's main objective was considered to be reporting the level of AI resource discrimination, along with different metrics' co‐occurrence. Great variability was found regarding utility assessment meanings and interpretations, even when authors were reporting the same metrics. Different AI resource users agreed on the lack of metrics' standardization when evaluating such resources and highlighted the need for research into AI resource evaluation and/or validation.

A clear increase was observed regarding the amount of relevant publications, involving a notable increase in web applications and generative models during 2023 and 2024. Developed countries accounted for most publications in this area, highlighting the opportunity for low‐ and middle‐income countries to contribute to this field, that is, producing more equitable knowledge and addressing disparities regarding technology and health outcomes [197].

Web applications were the most frequently reported resources in all studies consulted, reporting forms of comparison involving a wide range of metrics. The consistent inclusion of comparators reflected the ongoing need to build evidence supporting such resources' validation. Traditional human screening, often supported by benchmark databases, was found to be the most commonly used reference standard. However, there are still no clear guidelines for researchers regarding which characteristics an AI resource should meet to be considered reliable for its confident use [198, 200].

Most studies aimed at evaluating AI resource non‐inferiority compared to classical human screening seemed to think that they were similar to the diagnostic test field in a clinical setting [19]. Such analogy states that AI resources differentiate between two categories, as do clinical tests in health settings. This could explain why Sensitivity and Specificity were the most frequently reported metrics.

Unlike the clinical setting where categories are differentiated through predefined decision rules (i.e., when interpreting diagnostic test results), such differentiation regarding ML is based on statistical learning. This means that an algorithm learns from data patterns. If some categories are much more represented than others, an algorithm may not learn properly and can become biased, tending to favor the category which appears most often in the training data [200]. An ML‐based text classifier could have high specificity simply because it classifies almost everything as “not relevant” or appear very “sensitive” if it classifies almost everything as “relevant.” In other words, an algorithm may achieve seemingly good metrics without actually capturing a meaningful pattern [201]. Therefore, in scenarios involving category imbalance, it is recommended using metrics better reflecting the balance between both sides of any classification, such as F1‐score, precision–recall curves, or balanced accuracy, along with calibration measures or area under the curve (AUC) to represent overall performance [27].

Moreover, if metrics such as precision (or PPV) and/or recall (or Sensitivity) are interpreted, all false negatives are usually treated as equivalent. However, in practice, not all studies contribute equally to a final synthesis, especially regarding SLRs which study interventions. Missing a study containing extensive or unique data may meaningfully influence the conclusions or overall effect, whereas missing a study making a minimal contribution may have little effect. As a result, evaluating automated tools based solely on the amount of missed references may not fully reflect their real‐world impact. Approaches considering each study's relative contribution or weight would provide a more accurate assessment of false negatives' implications [19]. Although no clear body of knowledge has yet been developed regarding the ideal combination of metrics for evaluating an AI resource's validation for automate screening, a combination of measurements to cover different aspects of AI resources can be reported. Previous guidelines recommended that contingency table‐based metrics should be reported along with complementary metrics, jointly analyzing inclusions and exclusion in the same measurement [27].

Comparing studies' results can be challenging unless definitions have been harmonized, thereby highlighting the need for establishing standardized evaluation metrics for the automation tools used in SLRs. An example of this would be the concept of *cost* varying in different studies dealing with the same matter; one author might measure it as the additional percentage of documents which need to be screened to achieve a given level of estimated recall [101], whereas another might define it as the monetary price of a platform being evaluated, as expressed in USD [47].

There is a need to define the steps or phases which should be followed in this emerging field, similar to that which exists in the field of developing new diagnostic tests in a clinical setting. Most evaluations were conducted retrospectively, using already completed systematic reviews; the reported metrics thus mainly reflect how well an AI resource performed when evaluated using already existing data, thereby limiting its application in real‐world settings [119]. Moreover, some approaches use feature assessment as a methodology for identifying a specific technological tool's most valuable characteristics [18]; however, some studies have shown that AI resources' output seems to depend on a specific combination of data characteristics and an internal structure defining how an AI resource processes information in order to learn and make decisions [18]. A user's background can also affect assessment; what is considered an advantage or disadvantage will vary among reviewers depending on their context and objectives [184], thereby limiting feature assessment methodology's generalizability.

Perlman et al., have suggested key methods and results regarding process, outcome and structure measures, along with the pertinent statistics to prove improvement using automation. The authors offer a meaningful category of metrics based on a before–after design [119], presenting a valuable path for continuing to build/ensure metrics' standardization.

Insuk et al., have stressed that guidelines and training for researchers regarding the responsible use of AI resources should be developed [53]. However, it is not just a matter of turning on a light following guidelines, but about illuminating an entire path with a structured framework accompanying the whole process. A stepwise research program builds evidence gradually, beginning with exploratory studies and progressing to retrospective validation and, eventually, to evaluating whether a particular tool can reliably predict decisions and affect outcomes in real practice.

As SLRs form a pilar for EBM, involving integrative research, then there must be a guarantee that the SLR being produced do comply with expected quality standards. Some authors have even highlighted the need for including ethical oversight throughout the automation process; nevertheless, this did not emerge as a frequent category in our analysis [198]. Recent perspectives from Cochrane, along with Campbell Collaboration, JBI and the Collaboration for Environmental Evidence [202] have invited the responsible use of AI in evidence synthesis (RAISE).

### 4.1 Strength and Limitations

This review has synthesized a substantial body of evidence for identifying the shared characteristics of AI resources used for automated reference screening and summarized reported user experiences. Categories were defined for organizing study objectives, types of comparators, evidence from performance evaluations and authors' reflections. This work forms part of an ongoing research effort aimed at developing a methodological framework for helping researchers understand and anticipate what to expect when integrating AI resources into their data screening and how to assess the results after using them.

A key strength of this review lies in its comprehensive and structured approach, combining quantitative and qualitative information to provide a clear picture of a rapidly evolving field. By incorporating methodological and experiential insights, it offers a balanced understanding of how automation tools are being developed and used in practice.

Nonetheless, some limitations should be noted. Data extraction was limited to the names of metrics reported by the studies. This pragmatic approach was necessary because some studies reported metrics across numerous experiments, which made comprehensive extraction impractical. Previous reviews have meta‐analyzed metrics data; such analysis was beyond the scope of the present study. Moreover, our study identified the heterogeneity regarding the metrics' definitions used, studies objectives and reporting practices that reduce the comparability of results regarding such studies.

We did not collect or systematically evaluate information on validation practices across the included studies. Specifically, information regarding developers' independence during validation, the construction of the gold standard, and whether data sets were prospectively assembled or repurposed was not systematically extracted. This decision reflects the current state of the literature rather than an oversight: to the best of our knowledge, guidance on how to perform and report internal or external validation of AI resources for screening are non‐existent, and within AI contexts, validation processes are primarily formalized for clinical prediction models [200, 203], neither of which has been adopted in the specific context of AI resources for automate screening for systematic reviews.

## 5 Conclusions

The scoping review has identified gaps in both methodological approaches and the existing evidence base, particularly with regard to:

- Definition of study objectives and research phases in studies evaluating the automation of screening, to support consistent operationalisation across studies;
- Shared standards or benchmarks for comparing AI resources with other screening methodologies.
- The use of evaluation metrics, how they should be combined and how they should be interpreted.
- The predominant use of diagnostic test–based metrics (e.g., sensitivity and specificity) may be insufficient for evaluating AI screening tools, particularly under class imbalance.
- The underrepresentation of ethical oversight considerations in evaluations of screening automation, despite their relevance for ensuring quality and trustworthiness in evidence synthesis.

Future research could focus on:

- Development of theoretical and methodological frameworks to guide the design, conduct, interpretation of research in this field, enabling the adaptation or development of reporting guidelines, critical appraisal tools, and risk‐of‐bias assessments to support consistent and standardised use in practice
- Guidance on the relationship between data characteristics, model structure, and AI performance more transparent to users, supporting more informed decisions about the selection and use of screening tools
- Guidance on how to assess the maturity level of AI resources for automate screening in SLR

Although AI technologies will continue to evolve, maintaining a clear and consistent framework for interpreting research on AI resources can support understanding their level of maturity and facilitate informed decision‐making by users.

## Author Contributions

**Ana M. Barragán:** conceptualization, methodology, writing – original draft, formal analysis, writing – review and editing, validation, software, data curation, investigation, funding acquisition, visualization, project administration, resources. **Sara Elena Ortiz Bonett:** formal analysis, writing – review and editing, validation, investigation; writing – original draft; data curation. **Eliana‐Isabel Rodríguez‐Grande:** formal analysis, writing – review and editing, methodology. **Alvaro David Orjuela‐Cañón:** conceptualization, methodology, writing – review and editing, supervision, validation. **Oscar J. Perdomo:** methodology, software, supervision. **Guillermo Sánchez‐Vanegas:** conceptualization, methodology, writing – review and editing, validation.

## Conflicts of Interest

The authors declare no conflicts of interest.

## Supporting information


Supporting File

## Data Availability

The data that support the findings of this study are available from the corresponding author upon reasonable request. Data available at https://osf.io/yzsr8/.
