# Supplementary material for: Artificial Intelligence Resources for the Screening of Titles and Abstracts in Systematic Reviews: A Scoping Review
Source: Cochrane Evid Synth Methods. 2026 Aug 14;4(5):e70098. doi: 10.1002/cesm.70098 (PMC13489248; doi:10.1002/cesm.70098)
Supplement: Supplementary file 1 [file CESM-4-e70098-s001.zip › Appendix/Appendix 1. Final search strategy by database and date of search.docx]

**Appendix 1. Final search strategy by database and date of search**

1. PubMed

| **PCC question**  **component** | **Search algorithm** | **Results** | **Date** |
| --- | --- | --- | --- |
| Population | "Systematic Reviews as Topic"[Mesh] | 12,347 | 24/01/2024 |
|  | Systematic review[Title/Abstract] | 292,676 | 24/01/2024 |
|  | "Evidence-Based Medicine/methods"[Mesh] | 5,923 | 24/01/2024 |
|  | (Systematic review[Title/Abstract]) OR ("Evidence-Based Medicine/methods"[Mesh]) | 298,101 | 24/01/2024 |
|  | "Research Design"[Mesh] | 497,816 | 24/01/2024 |
|  | ((Systematic review[Title/Abstract]) OR ("Evidence-Based Medicine/methods"[Mesh])) OR ("Research Design"[Mesh]) | 779,412 | 24/01/2024 |
|  | ("Systematic Reviews as Topic"[Mesh]) OR (Systematic review[Title/Abstract]) | 295,994 | 24/01/2024 |
|  | ((("Systematic Reviews as Topic"[Mesh]) OR (Systematic review[Title/Abstract])) OR ("Evidence-Based Medicine/methods"[Mesh])) OR ("Research Design"[Mesh]) | 781,550 | 24/01/2024 |
|  | (((Systematic review[Title/Abstract]) OR ("Evidence-Based Medicine/methods"[Mesh])) OR ("Research Design"[Mesh])) OR ("Biomedical Research"[Mesh]) | 1,050,012 | 24/01/2024 |
|  | systematic review[Title/Abstract] | 292,710 | 24/01/2024 |
|  | review[Title/Abstract] | 2,252,121 | 24/01/2024 |
|  | systematic[Title/Abstract] | 572,691 | 24/01/2024 |
|  | (systematic[Title/Abstract]) AND (review[Title/Abstract]) | 348,502 | 24/01/2024 |
|  | ((((("Systematic Reviews as Topic"[Mesh]) OR ((systematic[Title/Abstract]) AND (review[Title/Abstract]))) OR (systematic review[Title/Abstract])) OR ("Research Design"[Mesh])) OR ("Evidence-Based Medicine/methods"[Mesh])) OR ("Biomedical Research"[Mesh]) | 1,102,374 | 24/01/2024 |
| Concept | "Algorithms"[Mesh] | 444,305 | 28/11/2023 |
|  | "machine learning"[Title/Abstract] | 100,183 | 28/11/2023 |
|  | "Data Mining"[Mesh] | 11,286 | 28/11/2023 |
|  | Artificial intelligence[Title/Abstract] | 42,535 | 28/11/2023 |
|  | Natural Language Processing[Title/Abstract] | 8,519 | 28/11/2023 |
|  | Data Mining[Title/Abstract] | 14,367 | 28/11/2023 |
|  | Deep learning[Title/Abstract] | 55,673 | 28/11/2023 |
|  | Supervised Machine Learning[Title/Abstract] | 2,925 | 28/11/2023 |
|  | Unsupervised Machine Learning[Title/Abstract] | 1,391 | 28/11/2023 |
|  | ("Algorithms"[Mesh]) OR ("machine learning"[Title/Abstract]) | 498,777 | 28/11/2023 |
|  | (("Algorithms"[Mesh]) OR ("machine learning"[Title/Abstract])) OR ("Data Mining"[Mesh]) | 505,193 | 28/11/2023 |
|  | ((("Algorithms"[Mesh]) OR ("machine learning"[Title/Abstract])) OR ("Data Mining"[Mesh])) OR (Artificial intelligence[Title/Abstract]) | 518,866 | 28/11/2023 |
|  | (((("Algorithms"[Mesh]) OR ("machine learning"[Title/Abstract])) OR ("Data Mining"[Mesh])) OR (Artificial intelligence[Title/Abstract])) OR (Natural Language Processing[Title/Abstract]) | 521,529 | 28/11/2023 |
|  | ((((("Algorithms"[Mesh]) OR ("machine learning"[Title/Abstract])) OR ("Data Mining"[Mesh])) OR (Artificial intelligence[Title/Abstract])) OR (Natural Language Processing[Title/Abstract])) OR (Data Mining[Title/Abstract]) | 529,442 | 28/11/2023 |
| Context | "Mass Screening"[Mesh] | 144,801 | 5/12/2023 |
|  | "Workload"[Mesh] | 24,095 | 5/12/2023 |
|  | "Classification"[Mesh] | 258,356 | 5/12/2023 |
|  | "Workflow"[Mesh] | 9,172 | 5/12/2023 |
|  | Mass screening[Title/Abstract] | 7,163 | 5/12/2023 |
|  | Workload[Title/Abstract] | 34,838 | 5/12/2023 |
|  | Workflow[Title/Abstract] | 37,288 | 5/12/2023 |
|  | Classification[Title/Abstract] | 435,052 | 5/12/2023 |
|  | ("Mass Screening"[Mesh]) OR (Mass screening[Title/Abstract]) | 147,723 | 5/12/2023 |
|  | ("Workload"[Mesh]) OR (Workload[Title/Abstract]) | 51,282 | 5/12/2023 |
|  | ("Classification"[Mesh]) OR (Classification[Title/Abstract]) | 680,508 | 5/12/2023 |
|  | ("Workflow"[Mesh]) OR (Workflow[Title/Abstract]) | 40,959 | 5/12/2023 |
|  | (("Mass Screening"[Mesh]) OR (Mass screening[Title/Abstract])) OR (("Workload"[Mesh]) OR (Workload[Title/Abstract])) | 198,528 | 5/12/2023 |
|  | ((("Mass Screening"[Mesh]) OR (Mass screening[Title/Abstract])) OR (("Workload"[Mesh]) OR (Workload[Title/Abstract]))) OR (("Classification"[Mesh]) OR (Classification[Title/Abstract])) | 875,609 | 5/12/2023 |
|  | (((("Mass Screening"[Mesh]) OR (Mass screening[Title/Abstract])) OR (("Workload"[Mesh]) OR (Workload[Title/Abstract]))) OR (("Classification"[Mesh]) OR (Classification[Title/Abstract]))) OR (("Workflow"[Mesh]) OR (Workflow[Title/Abstract])) | 913,330 | 5/12/2023 |
| Final | ((((((("Systematic Reviews as Topic"[Mesh]) OR ((systematic[Title/Abstract]) AND (review[Title/Abstract]))) OR (systematic review[Title/Abstract])) OR ("Research Design"[Mesh])) OR ("Evidence-Based Medicine/methods"[Mesh])) OR ("Biomedical Research"[Mesh])) AND ((((((((((("Algorithms"[Mesh]) OR ("machine learning"[Title/Abstract])) OR ("Data Mining"[Mesh])) OR (Artificial intelligence[Title/Abstract])) OR (Natural Language Processing[Title/Abstract])) OR (Data Mining[Title/Abstract])) OR (Deep learning[Title/Abstract])) OR (algorithm*[Title/Abstract])) OR ("Automation"[Mesh])) OR (automat*[Title/Abstract])) OR (("Software"[Mesh]) OR (software[Title/Abstract])))) AND ((((((("Workflow"[Mesh]) OR ((("Mass Screening"[Mesh]) OR ("Workload"[Mesh])) OR ("Classification"[Mesh]))) OR ("Databases as Topic"[Mesh])) OR ("Abstracting and Indexing"[Mesh])) OR ("Medical Informatics"[Mesh])) OR (screening[Title/Abstract])) OR (sampling[Title/Abstract])) Filters: from 2019 - 2024 | 8,943 | 25/01/2024 |

| **PCC question**  **component** | **Search algorithm** | **Results** | **Date** |
| --- | --- | --- | --- |
| First review | ((((((("Systematic Reviews as Topic"[Mesh]) OR ((systematic[Title/Abstract]) AND (review[Title/Abstract]))) OR (systematic review[Title/Abstract])) OR ("Research Design"[Mesh])) OR ("Evidence-Based Medicine/methods"[Mesh])) OR ("Biomedical Research"[Mesh])) AND ((((((((((("Algorithms"[Mesh]) OR ("machine learning"[Title/Abstract])) OR ("Data Mining"[Mesh])) OR (Artificial intelligence[Title/Abstract])) OR (Natural Language Processing[Title/Abstract])) OR (Data Mining[Title/Abstract])) OR (Deep learning[Title/Abstract])) OR (algorithm*[Title/Abstract])) OR ("Automation"[Mesh])) OR (automat*[Title/Abstract])) OR (("Software"[Mesh]) OR (software[Title/Abstract])))) AND ((((((("Workflow"[Mesh]) OR ((("Mass Screening"[Mesh]) OR ("Workload"[Mesh])) OR ("Classification"[Mesh]))) OR ("Databases as Topic"[Mesh])) OR ("Abstracting and Indexing"[Mesh])) OR ("Medical Informatics"[Mesh])) OR (screening[Title/Abstract])) OR (sampling[Title/Abstract])) Filters: from 2024 - 2025 | 1,919 | 19/02/2025 |

| **PCC question**  **component** | **Search algorithm** | **Results** | **Date** |
| --- | --- | --- | --- |
| Final | ((((((("Systematic Reviews as Topic"[Mesh]) OR ((systematic[Title/Abstract]) AND (review[Title/Abstract]))) OR (systematic review[Title/Abstract])) OR ("Research Design"[Mesh])) OR ("Evidence-Based Medicine/methods"[Mesh])) OR ("Biomedical Research"[Mesh])) AND ((((((((((("Algorithms"[Mesh]) OR ("machine learning"[Title/Abstract])) OR ("Data Mining"[Mesh])) OR (Artificial intelligence[Title/Abstract])) OR (Natural Language Processing[Title/Abstract])) OR (Data Mining[Title/Abstract])) OR (Deep learning[Title/Abstract])) OR (algorithm*[Title/Abstract])) OR ("Automation"[Mesh])) OR (automat*[Title/Abstract])) OR (("Software"[Mesh]) OR (software[Title/Abstract])))) AND ((((((("Workflow"[Mesh]) OR ((("Mass Screening"[Mesh]) OR ("Workload"[Mesh])) OR ("Classification"[Mesh]))) OR ("Databases as Topic"[Mesh])) OR ("Abstracting and Indexing"[Mesh])) OR ("Medical Informatics"[Mesh])) OR (screening[Title/Abstract])) OR (sampling[Title/Abstract])) Filters: from 02/2025 – 02/2026 | 2,400 | 28/02/2026 |

2. Embase

| **PCC question** **component** | **Search algorithm** | **Results** | **Date** |
| --- | --- | --- | --- |
| Final | #14 AND (2019:py OR 2020:py OR 2021:py OR 2022:py OR 2023:py OR 2024:py) | 162 | 19/02/2024 |
| Population AND Concept AND Context | #12 AND #13 | 237 | 19/02/2024 |
| Context | workload'/exp OR 'work load' OR 'workload' OR 'workload measurement' | 79,244 | 19/02/2024 |
| Population AND Concept | #10 AND #11 | 49,070 | 19/02/2024 |
| Population | systematic review (topic)'/exp OR 'systematic review (topic)' OR 'systematic reviews' OR 'systematic reviews as topic' | 136,812 | 19/02/2024 |
| Concept | data processing'/exp OR 'automation, computers and data processing' OR 'data processing' | 5,532,476 | 19/02/2024 |

| **PCC question component** | **Search algorithm** | **Results** | **Date** |
| --- | --- | --- | --- |
| First review | ('systematic review (topic)'/exp OR 'systematic review (topic)' OR 'systematic reviews' OR 'systematic reviews as topic') AND ('information processing'/exp OR 'data processing'/exp) AND ('workload'/exp OR 'work load' OR 'workload' OR 'workload measurement') AND (2024:py OR 2025:py) | 76 | 19/02/2025 |

| **PCC question component** | **Search algorithm** | **Results** | **Date** |
| --- | --- | --- | --- |
| Final | ('systematic review (topic)'/exp OR 'systematic review (topic)' OR 'systematic reviews' OR 'systematic reviews as topic') AND ('information processing'/exp OR 'data processing'/exp) AND ('workload'/exp OR 'work load' OR 'workload' OR 'workload measurement') AND [2025-2026]/py | 137 | 28/02/2026 |

3. Scopus

| **PCC question component** | **Search algorithm** | **Results** | **Date** |
| --- | --- | --- | --- |
| Final | ( TITLE-ABS-KEY ( algorithm ) ) AND ( TITLE-ABS ( title AND screening ) ) AND PUBYEAR > 2018 AND PUBYEAR < 2025 | 118 | 13/02/2024 |
| Concept AND Context | ( TITLE-ABS-KEY ( algorithm ) ) AND ( TITLE-ABS ( title AND screening ) ) | 153 | 13/02/2024 |
| Context | TITLE-ABS ( title AND screening ) | 6,527 | 13/02/2024 |
| Concept | TITLE-ABS-KEY ( algorithm ) | 4,014,834 | 13/02/2024 |

| **PCC question** c**omponent** | **Search algorithm** | **Results** | **Date** |
| --- | --- | --- | --- |
| First review | ( TITLE-ABS-KEY ( algorithm ) ) AND ( TITLE-ABS ( title AND screening ) ) AND PUBYEAR > 2024 | 55 | 19/02/2025 |

| **PCC question** c**omponent** | **Search algorithm** | **Results** | **Date** |
| --- | --- | --- | --- |
| Final | ( TITLE-ABS-KEY ( algorithm ) ) AND ( TITLE-ABS ( title AND screening ) ) AND PUBYEAR > 2025 | 101 | 28/02/2026 |

4. Web of science

| **PCC question component** | **Search algorithm** | **Filter** | **Results** | **Date** |
| --- | --- | --- | --- | --- |
| Final | (ALL=(algorithm )) AND ALL=(title AND abstract AND screening) | Refined By:Publication Years: 2019 or 2020 or 2021 or 2022 or 2023 or 2024 | 132 | 13/02/2024 |

| **PCC question component** | **Search algorithm** | **Filter** | **Results** | **Date** |
| --- | --- | --- | --- | --- |
| Final | (ALL=(algorithm )) AND ALL=(title AND abstract AND screening) | Refined By:Publication Years: 2024 or 2025 | 63 | 19/02/2025 |

| **PCC question component** | **Search algorithm** | **Filter** | **Results** | **Date** |
| --- | --- | --- | --- | --- |
| Final | (ALL=(algorithm )) AND ALL=(title AND abstract AND screening) | Refined By:Publication Years: 2025 or 2026 | 95 | 02/03/2026 |

5. IEE Xplore

| **PCC question component** | **Search algorithm** | **Filter** | **Results** | **Date** |
| --- | --- | --- | --- | --- |
| Final | ("All Metadata":algorithm) AND ("All Metadata":title AND "All Metadata":screening) | Filters Applied: 2019 - 2024 | 24 | 13/02/2024 |

| **PCC question component** | **Search algorithm** | **Filter** | **Results** | **Date** |
| --- | --- | --- | --- | --- |
| Final | ("All Metadata":algorithm) AND ("All Metadata":title AND "All Metadata":screening) | Filters Applied: 2024 - 2025 | 8 | 19/02/2025 |

| **PCC question component** | **Search algorithm** | **Filter** | **Results** | **Date** |
| --- | --- | --- | --- | --- |
| Final | ("All Metadata":algorithm) AND ("All Metadata":title AND "All Metadata":screening) | Filters Applied: 2025 - 2026 | 5 | 28/02/2026 |

6. Science Direct

| **PCC question component** | **Search algorithm** | **Filter** | **Results** | **Date** |
| --- | --- | --- | --- | --- |
| Final | (automation) AND ("title screening" OR "abstract screening") | Refine by 2019-2024 | 270 | 13/02/2024 |

| **PCC question component** | **Search algorithm** | **Filter** | **Results** | **Date** |
| --- | --- | --- | --- | --- |
| Final | (automation) AND ("title screening" OR "abstract screening") | Refined by 2024-2025 | 223 | 19/02/2025 |

| **PCC question component** | **Search algorithm** | **Filter** | **Results** | **Date** |
| --- | --- | --- | --- | --- |
| Final | (automation) AND ("title screening" OR "abstract screening") | Refined by 2025-2026 | 666 | 28/02/2026 |

7. Cochrane

| **PCC question component** | **Search algorithm** | **Filter** | **Results** | **Date** |
| --- | --- | --- | --- | --- |
| Final | title in Title Abstract Keyword AND abstract in Title Abstract Keyword AND screen* in Title Abstract Keyword - (Word variations have been searched) | 2019-2024 | 207 | 13/02/2024 |

| **PCC question component** | **Search algorithm** | **Filter** | **Results** | **Date** |
| --- | --- | --- | --- | --- |
| Final | title in Title Abstract Keyword AND abstract in Title Abstract Keyword AND screen* in Title Abstract Keyword - (Word variations have been searched) | 2024-2025 | 1 | 19/02/2025 |

| **PCC question component** | **Search algorithm** | **Filter** | **Results** | **Date** |
| --- | --- | --- | --- | --- |
| Final | title in Title Abstract Keyword AND abstract in Title Abstract Keyword AND screen* in Title Abstract Keyword - (Word variations have been searched) | 2025-2026 | 38 | 28/02/2026 |

8. Google Scholar

| **PCC question component** | **Search algorithm** | **Filter** | **Results** | **Date** |
| --- | --- | --- | --- | --- |
| Final | (Algorithm\|"artificial intelligence")(Title\|Abstract)(Repository)(Screen*) | 2019 - 2024 | 49 (first 5 pages) | 13/02/2024 |

| **PCC question component** | **Search algorithm** | **Filter** | **Results** | **Date** |
| --- | --- | --- | --- | --- |
| Final | (Algorithm\|"artificial intelligence")(Title\|Abstract)(Repository)(Screen*) | 2024 - 2025 | 65 (first 7 pages) | 19/02/2025 |

| **PCC question component** | **Search algorithm** | **Filter** | **Results** | **Date** |
| --- | --- | --- | --- | --- |
| Final | (Algorithm\|"artificial intelligence")(Title\|Abstract)(Repository)(Screen*) | 2025 - 2026 | 50 (first 5 pages) | 28/02/2026 |

9. Epistemonikos

| **PCC question component** | **Search algorithm** | **Filter** | **Results** | **Date** |
| --- | --- | --- | --- | --- |
| Final | (title:(title) OR abstract:(title)) OR (title:(abstract) OR abstract:(abstract)) AND (title:(screening) OR abstract:(screening)) AND (title:(algorithm) OR abstract:(algorithm)) | 2019-2024 | 26 | 13/02/2024 |

| **PCC question component** | **Search algorithm** | **Filter** | **Results** | **Date** |
| --- | --- | --- | --- | --- |
| Final | (title:(title) OR abstract:(title)) OR (title:(abstract) OR abstract:(abstract)) AND (title:(screening) OR abstract:(screening)) AND (title:(algorithm) OR abstract:(algorithm)) | 2024-2025 | 7 | 19/02/2025 |

| **PCC question component** | **Search algorithm** | **Filter** | **Results** | **Date** |
| --- | --- | --- | --- | --- |
| Final | (title:(title) OR abstract:(title)) OR (title:(abstract) OR abstract:(abstract)) AND (title:(screening) OR abstract:(screening)) AND (title:(algorithm) OR abstract:(algorithm)) | 2025-2026 | 11 | 28/02/2026 |
