# Supplementary material for: Artificial Intelligence Resources for the Screening of Titles and Abstracts in Systematic Reviews: A Scoping Review
Source: Cochrane Evid Synth Methods. 2026 Aug 14;4(5):e70098. doi: 10.1002/cesm.70098 (PMC13489248; doi:10.1002/cesm.70098)
Supplement: Supplementary file 1 [file CESM-4-e70098-s001.zip › Appendix/Appendix 2. References and reasons for exclusions regarding full-text evaluation.docx]

**Appendix 2. References and reasons for exclusions regarding full-text evaluation**

| **First author’s surname** | **Year** | **Reason for exclusion** | **Reference** |
| --- | --- | --- | --- |
| Paynter | 2021 | No AI algorithm was reported for title/abstract screening | Paynter, R. A., Fiordalisi, C., Stoeger, E., Erinoff, E., Featherstone, R., Voisin, C., & Adam, G. P. (2021). A Prospective Comparison of Evidence Synthesis Search Strategies Developed With and Without Text-Mining Tools. Agency for Healthcare Research and Quality (US) |
| van Dinter | 2021 | This was a preliminary version; we included the latest version | van Dinter, R., Catal, C., & Tekinerdogan, B. (2021). A Multi-Channel Convolutional Neural Network approach to automate the citation screening process. Applied Soft Computing, 112. https://doi.org/10.1016/j.asoc.2021.107765 |
| Whelan | 2021 | Out of the scope of the review | Whelan, J., Ghoniem, M., Médoc, N., Apicella, M., & Beck, E. (2021). Applying a novel approach to scoping review incorporating artificial intelligence: mapping the natural history of gonorrhoea. BMC Medical Research Methodology, 21(1), 183. https://doi.org/10.1186/s12874-021-01367-x |
| Amezcua-Prieto | 2020 | No AI algorithm was reported for title/abstract screening | Amezcua-Prieto, C., Fernández-Luna, J. M., Huete-Guadix, J. F., Bueno-Cavanillas, A., & Khan, K. S. (2020). Artificial intelligence and automation of systematic reviews in women’s health. In *Current Opinion in Obstetrics and Gynecology* (Vol. 32, Issue 5, pp. 335–341). Lippincott Williams and Wilkins. https://doi.org/10.1097/GCO.0000000000000643 |
| Zhang | 2022 | Results not reported | Zhang, Y., Liang, S., Feng, Y., Wang, Q., Sun, F., Chen, S., Yang, Y., He, X., Zhu, H., & Pan, H. (2022). Automation of literature screening using machine learning in medical evidence synthesis: a diagnostic test accuracy systematic review protocol. In Systematic Reviews (Vol. 11, Issue 1). BioMed Central Ltd. https://doi.org/10.1186/s13643-021-01881-5 |
| Noel-Storr | 2020 | No AI algorithm was reported for title/abstract screening | Noel-Storr, A. H., Dooley, G., Wisniewski, S., Glanville, J., Thomas, J., Cox, S., Featherstone, R., & Foxlee, R. (2020). Cochrane Centralised Search Service showed high sensitivity identifying randomised controlled trials: A retrospective analysis. Journal of clinical epidemiology, 127, 142–150. https://doi.org/10.1016/j.jclinepi.2020.08.008 |
| Sampson | 2021 | Out of the scope of the review | Sampson, M., Nama, N., O’Hearn, K., Murto, K., Nasr, A., Katz, S. L., Macartney, G., Momoli, F., & McNally, J. D. (2020). Creating enriched training sets of eligible studies for large systematic reviews: the utility of PubMed’s Best Match algorithm. International Journal of Technology Assessment in Health Care, 37, e7. https://doi.org/10.1017/S0266462320002159 |
| Wang | 2020 | No AI algorithm was reported for title/abstract screening | Wang, Z., Nayfeh, T., Tetzlaff, J., O’Blenis, P., & Murad, M. H. (2020). Error rates of human reviewers during abstract screening in systematic reviews. In *PLOS ONE* (Vol. 15, Issue 1). Public Library of Science. https://doi.org/10.1371/journal.pone.0227742 |
| O'Hearn | 2021 | No AI algorithm was reported for title/abstract screening | O’Hearn, K., MacDonald, C., Tsampalieros, A., Kadota, L., Sandarage, R., Jayawarden, S. K., Datko, M., Reynolds, J. M., Bui, T., Sultan, S., Sampson, M., Pratt, M., Barrowman, N., Nama, N., Page, M., & McNally, J. D. (2021). Evaluating the relationship between citation set size, team size and screening methods used in systematic reviews: a cross-sectional study. *BMC Medical Research Methodology*, *21*(1). https://doi.org/10.1186/s12874-021-01335-5 |
| Dunn | 2020 | No AI algorithm was reported for title/abstract screening | Dunn, A. G., & Bourgeois, F. T. (2020). Is it time for computable evidence synthesis? In *Journal of the American Medical Informatics Association* (Vol. 27, Issue 6, pp. 972–975). Oxford University Press. https://doi.org/10.1093/jamia/ocaa035 |
| Thiabaud | 2020 | No AI algorithm was reported for title/abstract screening | Thiabaud, A., Triulzi, I., Orel, E., Tal, K., & Keiser, O. (2020). Social, behavioral, and cultural factors of HIV in Malawi: Semi-automated systematic review. In *Journal of Medical Internet Research* (Vol. 22, Issue 8). JMIR Publications Inc. https://doi.org/10.2196/18747 |
| van Haastrecht | 2021 | Out of the scope of the review | van Haastrecht, M., Sarhan, I., Yigit Ozkan, B., Brinkhuis, M., & Spruit, M. (2021). SYMBALS: A Systematic Review Methodology Blending Active Learning and Snowballing. Frontiers in research metrics and analytics, 6, 685591. https://doi.org/10.3389/frma.2021.685591 |
| Scott | 2021 | Out of the scope of the review | Scott, A. M., Forbes, C., Clark, J., Carter, M., Glasziou, P., & Munn, Z. (2021). Systematic review automation tools improve efficiency but lack of knowledge impedes their adoption: a survey. Journal of clinical epidemiology, 138, 80–94. https://doi.org/10.1016/j.jclinepi.2021.06.030 |
| Escaldelai | 2022 | No AI algorithm was reported for title/abstract screening | Escaldelai, F. M. D., Escaldelai, L., & Bergamaschi, D. P. (2022). Systematic Review Support software system: web-based solution for managing duplicates and screening eligible studies. *Revista Brasileira de Epidemiologia*, *25*. https://doi.org/10.1590/1980-549720220030 |
| Bouhadana | 2022 | Out of the scope of the review | Bouhadana, D., Lu, X. H., Luo, J. W., Assad, A., Deyirmendjian, C., Guennoun, A., Nguyen, D. D., Kwong, J. C. C., Chughtai, B., Elterman, D. S., Zorn, K. C., Trinh, Q. D., & Bhojani, N. (2023). Clinical Applications of Machine Learning for Urolithiasis and Benign Prostatic Hyperplasia: A Systematic Review. Journal of Endourology. https://doi.org/10.1089/end.2022.0311 |
| Blaizot | 2021 | Systematic review on tools for automating systematic reviews in general | Blaizot, A., Veettil, S. K., Saidoung, P., Moreno-Garcia, C. F., Wiratunga, N., Aceves-Martins, M., Lai, N. M., & Chaiyakunapruk, N. (2022). Using artificial intelligence methods for systematic review in health sciences: A systematic review. Research Synthesis Methods, 13(3), 353–362. https://doi.org/10.1002/jrsm.1553 |
| Surian | 2021 | Out of the scope of the review | Surian, D., Bourgeois, F. T., & Dunn, A. G. (2021). The automation of relevant trial registration screening for systematic review updates: an evaluation study on a large dataset of ClinicalTrials.gov registrations. BMC Medical Research Methodology, 21(1), 281. https://doi.org/10.1186/s12874-021-01485-6 |
| Witzmann | 2022 | No AI algorithm was reported for title/abstract screening | Witzmann, A., Queiros, L., & Abogunrin, S. (2022). P59 Automated Literature Screening Using Artificial Intelligence: A Meta-Analysis of Different Algorithm Results. Value in Health, 25(12, Supplement), S14. https://doi.org/https://doi.org/10.1016/j.jval.2022.09.07 |
| Thomas | 2021 | Out of the scope of the review | Thomas, J., McDonald, S., Noel-Storr, A., Shemilt, I., Elliott, J., Mavergames, C., & Marshall, I. J. (2021). Machine learning reduced workload with minimal risk of missing studies: development and evaluation of a randomised controlled trial classifier for Cochrane Reviews. Journal of clinical epidemiology, 133, 140–151. https://doi.org/10.1016/j.jclinepi.2020.11.003 |
| van Altena | 2019 | No AI algorithm was reported for title/abstract screening | van Altena, A. J., Spijker, R., & Olabarriaga, S. D. (2019). Usage of automation tools in systematic reviews. Research synthesis methods, 10(1), 72–82. https://doi.org/10.1002/jrsm.1335 |
| Queiros | 2022 | No AI algorithm was reported for title/abstract screening | Queiros, L., Witzmann, A., Sumner, M., Wehler, P., Baehrens, D., & Abogunrin, S. (2022). POSB317 Machines As a Second Reviewer in Systematic Literature Reviews. *Value in Health*, *25*(1), S205–S206. https://doi.org/10.1016/j.jval.2021.11.1000 |
| Scott | 2023 | No AI algorithm was reported for title/abstract screening | Scott, A. M., Glasziou, P., & Clark, J. (2023a). We extended the 2-week systematic review (2weekSR) methodology to larger, more complex systematic reviews: A case series. *Journal of Clinical Epidemiology*, *157*, 112–119. https://doi.org/10.1016/j.jclinepi.2023.03.007 |
| Scott | 2023 | The machine learning algorithm has not been reported | Scott, A. M., Glasziou, P., & Clark, J. (2023b). We extended the 2-week systematic review (2weekSR) methodology to larger, more complex systematic reviews: A case series. *Journal of Clinical Epidemiology*, *157*, 112–119. https://doi.org/10.1016/j.jclinepi.2023.03.007 |
| Halfpenny | 2021 | No AI algorithm was reported for title/abstract screening | Halfpenny, N., Alleman, C., Eaton, J., & van Vliet, M. (2019). Using machine learning for efficiency improvements in systematic literature reviews of clinical efficacy and safety. *Value in Health*, *22*, s821. |
| Tufanaru | 2024 | Out of the scope of the review | Tufanaru, C., Surian, D., Scott, A. M., Glasziou, P., & Coiera, E. (2024). The 2-week systematic review (2weekSR) method was successfully blind-replicated by another team: a case study. *Journal of Clinical Epidemiology*, *165*. https://doi.org/10.1016/j.jclinepi.2023.10.013 |
| Hair | 2023 | Out of the scope of the review | Hair, K., Wilson, E., Wong, C., Tsang, A., Macleod, M., & Bannach-Brown, A. (2023). Systematic online living evidence summaries: emerging tools to accelerate evidence synthesis. Clinical Science (London, England : 1979), 137(10), 773–784. https://doi.org/10.1042/CS20220494 |
| Haby | 2024 | No AI algorithm was reported for title/abstract screening | Haby, M. M., Barreto, J. O. M., Kim, J. Y. H., Peiris, S., Mansilla, C., Torres, M., Guerrero-Magaña, D. E., & Reveiz, L. (2024). What are the best methods for rapid reviews of the research evidence? A systematic review of reviews and primary studies. Research synthesis methods, 15(1), 2–20. https://doi.org/10.1002/jrsm.1664 |
| Tetzlaff | 2019 | Out of the scope of the review | Tetzlaff, J., Cadarette, S. M., O’Blenis, P., & Ruiz, K. (2019). PNS15 PRAGMATIC ARTIFICIAL INTELLIGENCE-BASED REFERENCE SCREENING IN SYSTEMATIC REVEIWS. ARE TWO ROBOTS BETTER THAN ONE? *Value in Health*, *22*, S290. https://doi.org/10.1016/j.jval.2019.04.1381 |
| Mozgai | 2023 | Out of the scope of the review | Mozgai, S., Kaurloto, C., Winn, J., Leeds, A., Heylen, D., Hartholt, A., & Scherer, S. (2023). Machine learning for semi-automated scoping reviews. Intelligent Systems with Applications, 19, 200249. https://doi.org/10.1016/j.iswa.2023.200249 |
| Marshall | 2023 | Out of the scope of the review | Marshall, I. J., Trikalinos, T. A., Soboczenski, F., Yun, H. S., Kell, G., Marshall, R., & Wallace, B. C. (2023). In a pilot study, automated real-time systematic review updates were feasible, accurate, and work-saving. Journal of Clinical Epidemiology, 153, 26–33. https://doi.org/10.1016/j.jclinepi.2022.08.013 |
| Brockmeier | 2019 | No AI algorithm was reported for title/abstract screening | Brockmeier, A. J., Ju, M., Przybyła, P., & Ananiadou, S. (2019). Improving reference prioritisation with PICO recognition. BMC medical informatics and decision making, 19(1), 256. https://doi.org/10.1186/s12911-019-0992-8 |
| Robson | 2019 | No AI algorithm was reported for title/abstract screening | Robson, R. C., Pham, B., Hwee, J., Thomas, S. M., Rios, P., Page, M. J., & Tricco, A. C. (2019). Few studies exist examining methods for selecting studies, abstracting data, and appraising quality in a systematic review. In *Journal of Clinical Epidemiology* (Vol. 106, pp. 121–135). Elsevier USA. https://doi.org/10.1016/j.jclinepi.2018.10.003 |
| Ierardi | 2023 | No AI algorithm was reported for title/abstract screening | Ierardi, E., Eilbeck, J. C., van Wijck, F., Ali, M., & Coupar, F. (2023). Data mining versus manual screening to select papers for inclusion in systematic reviews: A novel method to increase efficiency. *International Journal of Rehabilitation Research*, *46*(3), 284–292. https://doi.org/10.1097/MRR.0000000000000595 |
| Afifi | 2023 | No AI algorithm was reported for title/abstract screening | Afifi, M., Stryhn, H., & Sanchez, J. (2023). Data extraction and comparison for complex systematic reviews: a step-by-step guideline and an implementation example using open-source software. *Systematic Reviews*, *12*(1). https://doi.org/10.1186/s13643-023-02322-1 |
| Nama | 2019 | No AI algorithm was reported for title/abstract screening | Nama, N., Sampson, M., Barrowman, N., Sandarage, R., Menon, K., Macartney, G., Murto, K., Vaccani, J. P., Katz, S., Zemek, R., Nasr, A., & McNally, J. D. (2019). Crowdsourcing the citation screening process for systematic reviews: Validation study. In *Journal of Medical Internet Research* (Vol. 21, Issue 4). JMIR Publications Inc. https://doi.org/10.2196/12953 |
| Roberts | 2023 | Out of the scope of the review | Roberts, R. H. R., Ali, S. R., Hutchings, H. A., Dobbs, T. D., & Whitaker, I. S. (2023). Comparative study of ChatGPT and human evaluators on the assessment of medical literature according to recognised reporting standards. BMJ Health & Care Informatics, 30(1), e100830. https://doi.org/10.1136/bmjhci-2023-100830 |
| Kanoulas | 2019 | No AI algorithm was reported for title/abstract screening | Kanoulas, E., Li, D., Azzopardi, L., & Spijker, R. (n.d.). *Reviews in Empirical Medicine Overview*. |
| Šuster | 2023 | Out of the scope of the review | Šuster, S., Baldwin, T., Lau, J. H., Yepes, A. J., Iraola, D. M., Otmakhova, Y., & Verspoor, K. (2023). Automating Quality Assessment of Medical Evidence in Systematic Reviews: Model Development and Validation Study. J. Med. Internet Res., 25. https://doi.org/10.2196/35568 |
| Norman | 2018 | Outside the observation period (2018) | Norman, C., Leeflang, M., Zweigenbaum, P., & Névéol, A. (n.d.). Automating Document Discovery in the Systematic Review Process: How to Use Chaff to Extract Wheat. https://doi.org/10.5281/zenodo.1173076 |
| Lerner | 2018 | Outside the observation period (2018) | Lerner, I., Créquit, P., Ravaud, P., & Atal, I. (2019). Automatic screening using word embeddings achieved high sensitivity and workload reduction for updating living network meta-analyses. *Journal of Clinical Epidemiology*, *108*, 86–94. https://doi.org/10.1016/j.jclinepi.2018.12.001 |
| Orel | 2023 | No AI algorithm was reported for title/abstract screening | Orel, E., Ciglenecki, I., Thiabaud, A., Temerev, A., Calmy, A., Keiser, O., & Merzouki, A. (2023). An Automated Literature Review Tool (LiteRev) for Streamlining and Accelerating Research Using Natural Language Processing and Machine Learning: Descriptive Performance Evaluation Study. *Journal of Medical Internet Research*, *25*(1). https://doi.org/10.2196/39736 |
| Li | 2019 | Out of the scope of the review | Li, T., Saldanha, I. J., Jap, J., Smith, B. T., Canner, J., Hutfless, S. M., Branch, V., Carini, S., Chan, W., de Bruijn, B., Wallace, B. C., Walsh, S. A., Whamond, E. J., Murad, M. H., Sim, I., Berlin, J. A., Lau, J., Dickersin, K., & Schmid, C. H. (2019). A randomised trial provided new evidence on the accuracy and efficiency of traditional vs. electronically annotated abstraction approaches in systematic reviews. Journal of Clinical Epidemiology, 115, 77–89. https://doi.org/10.1016/j.jclinepi.2019.07.005 |
| Norman | 2019 | No AI algorithm was reported for title/abstract screening | Norman, C. R., Gargon, E., Leeflang, M. M. G., Névéol, A., & Williamson, P. R. (2019). Evaluation of an automatic article selection method for timelier updates of the Comet Core Outcome Set database. Database : the journal of biological databases and curation, 2019, baz109. https://doi.org/10.1093/database/baz109 |
| Affengruber | 2024 | Out of the scope of the review | Affengruber, L., van der Maten, M.M., Spiero, I. et al. An exploration of available methods and tools to improve the efficiency of systematic review production: a scoping review. BMC Med Res Methodol 24, 210 (2024). https://doi.org/10.1186/s12874-024-02320-4 |
| Masoumi | 2024 | Out of the scope of the review | Masoumi S, Amirkhani H, Sadeghian N, Shahraz S. Natural language processing (NLP) to facilitate abstract review in medical research: the application of BioBERT to exploring the 20-year use of NLP in medical research. Systematic Reviews. 2024 Dec 1;13(1). |
| Kovacevic | 2024 | Out of the scope of the review | Kovačević A, Bašaragin B, Milošević N, Nenadić G. De-identification of clinical free text using natural language processing: A systematic review of current approaches. Vol. 151, Artificial Intelligence in Medicine. Elsevier B.V.; 2024. |
| Abogunrin | 2025 | Out of the scope of the review | Abogunrin S, Muir JM, Zerbini C, Sarri G. How much can we save by applying artificial intelligence in evidence synthesis? Results from a pragmatic review to quantify workload efficiencies and cost savings. Vol. 16, Frontiers in Pharmacology. Frontiers Media SA; 2025. |
| Gao | 2024 | Out of the scope of the review | Gao, L., Cui, F., & Zhang, C. B. (2024). Library Similar Literature Screening System Research Based on LDA Topic Model. Journal of information & knowledge management, 23(5). https://doi.org/10.1142/S0219649224500771 |
| Oami | 2024 | Results not reported | Oami T, Okada Y, Nakada T. Comparison of large language models for citation screening: A protocol for a prospective study [Internet]. 2024. Available from: http://medrxiv.org/lookup/doi/10.1101/2024.06.26.24309513 |
| Liu | 2024 | Out of the scope of the review | Liu JJW, Ein N, Gervasio J, Easterbrook B, Nouri MS, Nazarov A, et al. Usability and agreement of the SWIFT-ActiveScreener systematic review support tool: Preliminary evaluation for use in clinical research. PLoS ONE. 2024 Nov 1;19(11). |
| Toth | 2024 | Out of the scope of the review | Tóth B, Berek L, Gulácsi L, Péntek M, Zrubka Z. Automation of systematic reviews of biomedical literature: a scoping review of studies indexed in PubMed. Systematic Reviews. 2024 Dec 1;13(1). |
| Pallath | 2023 | Out of the scope of the review | Pallath A, Zhang Q. Paperfetcher: A tool to automate handsearching and citation searching for systematic reviews. Research Synthesis Methods [Internet]. 2023 Mar 26;14(2):323–35. Available from: https://onlinelibrary.wiley.com/doi/10.1002/jrsm.1604 |
| Ma | 2022 | Out of the scope of the review | Ma, J., Wu, X., & Huang, L. (2022). The Use of Artificial Intelligence in Literature Search and Selection of the PubMed Database. Scientific Programming, 2022. https://doi.org/10.1155/2022/8855307 |
| Li | 2025 | Out of the scope of the review | Li, Y., Tian, J., Xu, A., Greiner, R., Hayward, J., Greenshaw, A. J., & Cao, B. (2025). Maturity Framework for Operationalizing Machine Learning Applications in Health Care: Scoping Review. In Journal of Medical Internet Research (Vol. 27). JMIR Publications Inc. https://doi.org/10.2196/66559 |
| Rogers | 2025 | Out of the scope of the review | Rogers, K., Miller, A., Girgis, A., Clark, E. C., Neil-Sztramko, S. E., & Dobbins, M. (2025). Leveraging AI to Optimize Maintenance of Health Evidence and Offer a One-Stop Shop for Quality-Appraised Evidence Syntheses on the Effectiveness of Public Health Interventions: Quality Improvement Project. Journal of Medical Internet Research, 27. https://doi.org/10.2196/69700 |
| Yao | 2025 | Out of the scope of the review | Yao, X., Low, A., Sivajohanathan, D., Vella, E., Wang, P., Kaur, G., Saha, A., & Sussman, J. (2025). Evaluation of artificial intelligence-based tool Covidence in literature screening for guideline updates: a prospective study. Intelligent Medicine. https://doi.org/10.1016/j.imed.2025.12.006 |
| Vallamchetla | 2025 | No AI algorithm was reported for title/abstract screening | Vallamchetla, S. K., Abdelkader, O., Elnaggar, A., Ramadan, D., Islam Shourav, M. M., Riaz, I. B., & Lin, M. P. (2025). Do it faster with PICOS: Generative AI-Assisted systematic review screening. Journal of Biomedical Informatics, 168. https://doi.org/10.1016/j.jbi.2025.104860 |
| Martin | 2025 | Results not reported | Martin, C. L., Bakker, C. J., Marquard, J. L., Sittig, S., Allain, J., Gopikrishnan, A. D., Chih, M. Y., Kim, K. K., Peta, V. J., & Gao, G. (2025). Transforming Literature Reviews in Healthcare Research with Artificial Intelligence: Preliminary Insights. Studies in Health Technology and Informatics, 329, 1648–1649. https://doi.org/10.3233/SHTI251145 |
| Courvoisier | 2025 | Out of the scope of the review | Courvoisier, D. S., Buitrago-Garcia, D., Buclin, C. P., Bürgisser, N., Iudici, M., & Mongin, D. (2025). Beyond human gold standards: A multimodel framework for automated abstract classification and information extraction. Research Synthesis Methods. https://doi.org/10.1017/rsm.2025.10054 |
| Robleto | 2025 | Results not reported | Robleto, E., & Shehadeh, L. A. (2025). Accelerating systematic reviews: a novel 1-wk screening protocol using rule-based automation with AI-assisted Python coding. American Journal of Physiology. Heart and Circulatory Physiology, 329(5), H1391–H1413. https://doi.org/10.1152/ajpheart.00374.2025 |
| Li | 2025 | Out of the scope of the review | Li, Y., Huang, Z., Luan, Z., Xu, S., Zhang, Y., Wu, L., Wu, D., Han, D., & Liu, Y. (2025). Efficient evidence selection for systematic reviews in traditional Chinese medicine. BMC Medical Research Methodology, 25(1). https://doi.org/10.1186/s12874-024-02430-z |
| Hasegawa | 2025 | Out of the scope of the review | Hasegawa, T., Kizaki, H., Ikegami, K., Imai, S., Yanagisawa, Y., Yada, S., Aramaki, E., & Hori, S. (2025). Improving Systematic Review Updates With Natural Language Processing Through Abstract Component Classification and Selection: Algorithm Development and Validation. JMIR Medical Informatics, 13. https://doi.org/10.2196/65371 |
| Hardy | 2024 | Out of the scope of the review | Hardy, E., Jenkins, A., Ross, J., & Lang, S. (2024). MSR229 Accuracy and Efficiency of Automated or Artificial Intelligence Tools in Systematic Literature Reviews: A Systematic Literature Review. *Value in Health*, *27*(12), S483. https://doi.org/10.1016/j.jval.2024.10.2462 |
| Mao | 2025 | Out of the scope of the review | Mao, X., Leelanupab, T., Scells, H., & Zuccon, G. (2025). *DenseReviewer: A Screening Prioritisation Tool for Systematic Review based on Dense Retrieval*. http://arxiv.org/abs/2502.03400 |
