# Supplementary material for: Artificial Intelligence Resources for the Screening of Titles and Abstracts in Systematic Reviews: A Scoping Review
Source: Cochrane Evid Synth Methods. 2026 Aug 14;4(5):e70098. doi: 10.1002/cesm.70098 (PMC13489248; doi:10.1002/cesm.70098)
Supplement: Supplementary file 1 [file CESM-4-e70098-s001.zip › Appendix/Appendix 3. Overview and temporal trends of datasets and AI resource use in screening studies (2019–2026).docx]

**Appendix 3. Overview and temporal trends of datasets and AI resource use in screening studies (2019–2026)**

a. **Overview of datasets used in AI resources reference screening studies 2019-2026. (Source: Authors’ elaboration)**

Figure code generated using Claude 4.6 Sonnet and rendered via Colab Phyton. Final layout edited and verified by the authors.

The figure summarizes the number and sample size of datasets employed to develop and evaluate AI resources.

b. **Dataset size vs. datasets per study & publication period. (Source: Authors’ elaboration)**

Figure code generated using Claude 4.6 Sonnet and rendered via Colab Phyton. Final layout edited and verified by the authors.

The figure illustrates the relationship between the size of datasets used and the number of datasets included per study, stratified by publication year.

c. **Temporal trends in AI resources screening studies 2019-2026. (Source: Authors’ elaboration)**

Figure code generated using Claude 4.6 Sonnet and rendered via Colab Phyton. Final layout edited and verified by the authors.

The figure presents the evolution of the median dataset size, illustrating annual changes in the volume datasets used to evaluate AI resources.
