# Supplementary material for: Artificial Intelligence Resources for the Screening of Titles and Abstracts in Systematic Reviews: A Scoping Review
Source: Cochrane Evid Synth Methods. 2026 Aug 14;4(5):e70098. doi: 10.1002/cesm.70098 (PMC13489248; doi:10.1002/cesm.70098)
Supplement: Supplementary file 1 [file CESM-4-e70098-s001.zip › Appendix/Appendix 4. Heatmap regarding type of AI resources by year..docx]

**Appendix 4. Heatmap regarding type of AI resources by year.**

a. **Web applications. (Source: Authors’ elaboration)**

Figure code generated using ChatGPT 3.5 and rendered via Google Colab (Python). Final layout edited and verified by the authors

* Covidence web-based programme optimised for Google Chrome, RMES = rapid medical evidence synthesis.

Distribution of different types of web applications found by year of publication

b. **Model comparison. (Source: Authors’ elaboration)**

Figure code generated using ChatGPT 3.5 and rendered via Google Colab (Python). Final layout edited and verified by the authors

Convolutional neural network = CNN, generalized linear models = GLM, Gated Recurrent Units = GRU, K-nearest neighbours = KNN, Gradient boosting = GB, Logistic regression with elastic net regularization = LogReg-EN, Long Short Term Memory = LSTM, Parallel convolutional neural network = PCNN, Parallel Stacked Convolutional Neural Network = PSCNN, Stacked convolutional neural network = SCNN

Distribution of the different types of model comparisons found by year of publication

c. **Generative models and Pre-trained models. (Source: Authors’ elaboration)**

Figure code generated using ChatGPT 3.5 and rendered via Google Colab (Python). Final layout edited and verified by the authors

Distribution of the different types of generative models and pre–trained models found by year of publication

d. **Pipeline/Workflow. (Source: Authors’ elaboration)**

Figure code generated using ChatGPT 3.5 and rendered via Google Colab (Python). Final layout edited and verified by the authors

Distribution of the different types of pipeline/workflows found by year of publication
