# Supplementary material for: Artificial Intelligence Resources for the Screening of Titles and Abstracts in Systematic Reviews: A Scoping Review
Source: Cochrane Evid Synth Methods. 2026 Aug 14;4(5):e70098. doi: 10.1002/cesm.70098 (PMC13489248; doi:10.1002/cesm.70098)
Supplement: Supplementary file 1 [file CESM-4-e70098-s001.zip › Appendix/Appendix 5. Comparing automation resources to other screening methodologies. (Source Authors’ elaboration).docx]

**Appendix 5. Comparing automation resources to other screening methodologies. (Source: Authors’ elaboration)**

| **Type of comparator for performance and utility evaluation** | **n** | **%** |
| --- | --- | --- |
| Retrospective comparison of results produced by human reviewers using a classical review process | 91 | 63 |
| Concurrent comparison to human reviewers performing a classical review | 42 | 29 |
| Concurrent comparison involving a single human reviewer | 9 | 6 |
| Comparing different stopping criteria for model calibration | 1 | 1 |
| Not specified | 1 | 1 |
| **Total** | **144** |  |
