# Supplementary material for: Artificial Intelligence Resources for the Screening of Titles and Abstracts in Systematic Reviews: A Scoping Review
Source: Cochrane Evid Synth Methods. 2026 Aug 14;4(5):e70098. doi: 10.1002/cesm.70098 (PMC13489248; doi:10.1002/cesm.70098)
Supplement: Supplementary file 1 [file CESM-4-e70098-s001.zip › Appendix/Appendix 6. Bar graph regarding type of assessment by resource category. (Source Authors’ elaboration).docx]

**Appendix 6. Bar graph regarding type of assessment by resource category. (Source: Authors’ elaboration)**

Figure code generated using ChatGPT 3.5 and rendered via Google Colab (Python). Final layout edited and verified by the authors

Frequency distribution regarding type of assessment by resource category
