# Supplementary material for: Artificial Intelligence Resources for the Screening of Titles and Abstracts in Systematic Reviews: A Scoping Review
Source: Cochrane Evid Synth Methods. 2026 Aug 14;4(5):e70098. doi: 10.1002/cesm.70098 (PMC13489248; doi:10.1002/cesm.70098)
Supplement: Supplementary file 1 [file CESM-4-e70098-s001.zip › Appendix/Appendix 7. How the metrics reported regarding Criterion assessment have been defined in the relevant papers selected here. (Source Author.docx]

**Appendix 7. How the metrics reported regarding Criterion assessment have been defined in the relevant papers selected here. (Source: Authors’ elaboration)**

| **Category** | **Metric** | **Description** |
| --- | --- | --- |
| Accuracy | S | - The proportion of citations correctly predicted as relevant by the tool out of the total deemed relevant by the human screener ^16,18,28,31,34–37,39,44,45,50,53,54,57,60,61,64,71,72,74,77,81–83,90,97–99,106,109,111,115,118,122,124,126,127,129,130,134,137,138,140,141,143,144,148,150–155,165,169–172^ .   Sensitivity = (true positives)/(true positives + false negatives) |
|  | Sp | - The proportion of citations correctly predicted as irrelevant by the tool out of the total deemed irrelevant by the human screener ^16,18,28,31,34–37,39,43–45,50,54,57,60,61,63,64,68,70–74,77,81–83,87,90,93,97,106,109,111,115,118,122,124,126,127,129,130,134,138,140,141,143,144,148,150,152–155,165,169–172^.   Specificity = (true negatives)/(true negatives + false positives) |
|  | Recall | - The proportion of citations correctly predicted as relevant by the tool out of the total deemed relevant by the human screener^29,31,32,38,40,41,43,44,51,52,58,63,66–70,73,75,78–80,84,86–88,92,93,95,101,102,107,119,147,149,158–163,166,167,169,175–177^.   Recall = (true positives)/(true positives + false negatives) |
|  | Accuracy | - Number of true positives + true negatives divided by total number of articles^29,34,35,43,45,47,49,53,54,57,61,63,64,70–72,79–81,84,86,87,92,93,106,107,109,111,115,117,121,124–126,139,140,143,144,148,154,161,172,176^. - Number of abstracts included by human reviewer and tool/total number of abstracts screened^151^ . |
|  | AUROC | - Overall ability of the model to discriminate between relevant and irrelevant records. An AUC of 1.0 indicates perfect discrimination, whereas an AUC of 0.5 represents performance equivalent to random guessing^16,28,39,63,84,100,111,141,144,149,164,168,171,172,174^. |
|  | F1 score | - $2\times\frac{precision \times recall}{precision+recall}$ ^16,29,31,34,40,43–45,53,57,58,63,70,72,75,78,79,83,84,87,102,107,109,115,119,123,127,147,151,154,161–163,172,176,177^. |
|  | F2 score | - $\frac{5\times precision\times recall}{4\times precision+recall}$ ^70^ |
|  | Detection rate | - The proportion of all cases that are correctly identified as positive (true positives) by the LLM^31, 44^. |
|  | Detection prevalence | - The proportion of all cases that are predicted as positive by the LLM, regardless of their true status^31, 44^. |
|  | Balanced accuracy | - The average of sensitivity and specificity, reflecting performance equally across positive and negative classes ^31,39,44,73,83,90^. |
|  | Raw agreement | - $\frac{TP+TN}{N}$ ^73^. |
|  | False negatives (FN) | - The number of records that were deemed irrelevant by the tool that the human reviewers deemed relevant ^34,36,39,43,60,61,63,68,70,71,83,84,87,93,95,97,114,124,125,127,143,153,155,161,165^. |
|  | True negatives (TN) | - Number of records correctly identified as ineligible^34,36,39,60,61,63,68,70,71,83,84,87,93,112,124,125,127,143,153,155,157,161,165^. |
|  | False negative rate (FNR) | - The proportion of citations that were incorrectly predicted as irrelevant by the tool, out of the total number of citations deemed relevant by the human screener ^16,38,46,47,49,68,91,124,129,130,134,144,150,157,177^. |
|  | True positives (TP) | - Number of records correctly identified as eligible^34,36,39,55,60,61,63,68,70,71,83,84,87,93,112,114,124,125,127,143,153,155,157,161,165,177^. |
|  | False positives (FP) | - Number of records incorrectly identified as eligible^34,36,39,43,60,61,63,68,70,71,83,84,87,93,95,124,125,127,143,153,155,157,161,165,177^. |
|  | Consistency rate | - Variability in the repeated screening results of the same datasets across different rounds^60^.   $\frac{\# agreements on inclusion +\# agreements on exclusion}{total number of citations}$ |
|  | Consistency rate of exclusion rationales | - Degree of agreement between the exclusion reasons generated by an automated screening tool and those assigned by human reviewers (gold standard)^86^. |
|  | Chance hit rate | - $\left[ \left( Sensitivity\cdot Prevalence \right)+(Specificity\cdot(1-Prevalence) \right]$ ^126^. |
|  | Spearman correlation | - Quantify the degree of agreement between the rankings produced by different models, assessing how similarly they order documents in terms of relevance^80, 160^. |
|  | Article exclusión rate | - Total number of articles automatically excluded during a step divided by the total number of articles at the beginning^46^. |
|  | Proportion missed | - The proportion of citations incorrectly predicted as irrelevant by the tool that were included in the final evidence base, out of the total number of citations included in the final evidence base ^9,110,114,129,130,148^ . - Titles incorrectly predicted as irrelevant / all titles predicted as irrelevant ^124,134,144^. |
|  | Excluded | - Number of articles excluded by the tool^135^. |
|  | False positive rate (FPR) | - The proportion of citations that were incorrectly predicted as relevant by the tool, out of the total number of citations deemed relevant by the human screener^33,38,47,49,68,91,150,157^ . |
|  | Fbeta score | - Metric used in machine learning, particularly in classification tasks, to evaluate the effectiveness of a classifier in terms of both precision and recall. Fbeta = ((1 + beta^2) * Precision * Recall) / (beta^2 * Precision + Recall) ^72,^ ^149^ |
|  | Articles excluded by the filter (c) | - The number of PubMed-cited articles with an abstract analyzed using RMES and subsequently excluded after applying the indicated filter^145^. |
|  | Articles included in SR (b) | - The number of PubMed-cited articles used by the authors in their SR^145^. |
|  | Articles incorrectly excluded by the filter (d) | - The number of articles that were excluded by the indicated filter, but were included by the authors in their SR^145^. |
|  | Balance | - $\frac{True positives+False positives}{True negatives+False negatives}$ ^143^. |
|  | cAutonomy | - Determined by the proportion of records which were sent to the crowd resolver for a final decision. No of records not requiring resolution/total number of records in dataset^8^. |
|  | Cohen’s kappa | - Statistic between the aggregated human team’s list and each AI implementation’s result ^43,57,70,71,124,125,157^. |
|  | Fleiss’ Kappa | - Overall agreement across all five chatbots^57, 71^. |
|  | Correct reason for exclusion | - The number of citations whose true reason for exclusion fell above the 90% threshold over the total number of citations with any reason for exclusion^111^. |
|  | Cosine similarity | - Measures the cosine of the angle between two vectors of an inner product space. We were Interested in the similarity between reviews, and not between individual documents in these reviews. We therefore calculated the similarity based on the mean feature vector for all documents in each review ^159^. |
|  | Crowd Sensitivity (cS) | - Determined by the crowd’s ability to collectively correctly identify the records that were for inclusion within the review. TP/TP+FN^8^. |
|  | Crowd Specificity (cSp) | - Determined by the crowd’s ability to collectively correctly identify the records that were not relevant to the review. TN/TN+FP^8^. |
|  | Mean proportion of misclassified articles | - Randomly sampling 100 articles from the full set of articles marked ‘not relevant’ by the LitQuest (and not screened by the research assistant team) after reaching the LitQuest stop criteria, and checking that each was appropriately classified^85^. |
|  | Human error rates | - Proportion of relevant references that were incorrectly excluded by human reviewers during manual screening ^116^. |
|  | Included | - Articles included by the tool for full text screening^135^. |
|  | Macro F1-score | - The average of the F1 scores computed independently for each class, giving equal weight to all classes^121^. $\frac{1}{N}\sum_{i=1}^{N} F1i$ |
|  | Matthews correlation coefficient (MCC) | - Measure of the quality of classifications of a two-class classification model. Its value ranges from 1 to (+ 1) ^70,77,84,166^.   $MCC=\frac{TP\times TN-FP\times FN}{\sqrt{(TP+FP)(TP+FN)(TN+FP)(TN+FN)}}$ |
|  | Maybe | - Number of articles labeled by the tool as maybe that passed to full text screening for further reading^135^. |
|  | Mean average Precisión (MAP) | - A summary metric that averages the precision values obtained each time a relevant record is retrieved across all rankings. It evaluates how consistently the AI model ranks relevant studies near the top of the list, combining both precision and ranking quality into a single measure^80,160,173^. |
|  | Misses (number of missed relevant studies) | - Studies missed by the models ^30,171^ . |
|  | NLP error rates | - Proportion of relevant references that were incorrectly excluded by the natural language processing (NLP) classifier from the total number of references^116^. |
|  | Normalized number | - The part of the dataset that does not need additional screening, divided by the total number of records prior to being add as prior knowledge^108^. |
|  | Number of missed studies | - Number of eligible studies (according to the human reference standard) that were not identified by the automated workflow during abstract screening^115^. - To evaluate relevance, changes in the study pool and a subsequent potential change to the HTA conclusion were assessed for each outcome. Firstly, for all outcomes to which the missed study contributed data, we checked whether the estimated effects would change from significant to non-significant or vice versa if the study was omitted in the analysis. Secondly, we evaluated whether any changes in effects would change the HTA conclusion. If no such change was found for any outcome, the studies missed in the selection process were classified as “not relevant”^137^. |
|  | Number of relevant publications | - “Relevant” means either that all studies of the reference standard were identified or that the studies not identified were irrelevant to the HTA conclusion. “Relevant to the HTA conclusion” means that the overall conclusion about the benefit of a treatment option would not change^137^. |
|  | Number needed to read | - 1/precision^119^ |
|  | Percentage documents Screened | - $\frac{True Positive+False positive}{N}$ ^101^ |
|  | Prioritization Accuracy | - Proportion of records that must be screened, according to the tool’s predicted relevance order, to retrieve all studies ultimately included in the review (the “screening burden”). A higher prioritization accuracy indicates that relevant studies appear earlier in the ranked list, thereby minimizing manual screening effort^99^. |
|  | Proportion of conflicts | - number of conflicts between human reviewers and DistillerAI, which had to be resolved by a second human reviewer/ number of unscreened records^141^. |
|  | Proportion of correct rejections | - The proportion of references that were correctly identified as irrelevant by the tool, relative to all references that were truly irrelevant^156^. |
|  | Proportion of correct selections | - The proportion of references that were correctly identified as relevant by the tool, relative to all references that were truly relevant^156^. |
|  | Proportion of included abstracts | - Number of included abstracts/number of unscreened records after completion of the training set^141^. |
|  | Proportion of incorrect rejections | - The proportion of references incorrectly excluded by the tool that were actually relevant^156^. |
|  | Proportion of incorrect selections | - The proportion of references incorrectly identified as relevant by the tool, among all truly irrelevant references^156^. |
|  | Rank position of the last relevant document (L_Rel) | - The ordinal position of the last relevant record within the ranked list produced by the AI tool. It reflects how early all relevant studies appear in the screening order, lower values indicate better prioritization performance and more efficient retrieval of relevant references^173^. |
|  | S of excluded papers | - Measure of excluding irrelevant papers^121^. |
|  | S of included papers | - Measure of correctly identify relevant papers^121^. |
|  | Selection success rate | - The percentage of articles that were retained by the indicated RMES filter out of the total number of eligible articles. The denominator was the number of PubMed-cited articles included in the SR by the authors. A value of 100% indicates that the RMES filter correctly identified all of the PubMed-cited articles with an abstract that were used by the authors in their SR. A value of 0% indicates that the RMES filter did not retain any of those articles^145^. |
|  | True negative rate (TNR) | - Proportion of correctly excluded papers among all excluded papers ^49,174^. |
|  | True positive rate (TPR) | - Proportion of correctly included papers among all included papers ^49,174^. |
|  | True recall @95% | - [title/abstract TP / (title/abstract TP + title/abstract FN)]^112^. |
|  | Youden`s index and threshold | - A metric that combines sensitivity and specificity to identify the threshold that maximizes the overall performance of a classification model^144^. |
|  | Kappa | - Kappa statistics and 95% CIs were used to determine the level of agreement between the reviewer and the tool^16,83,90,121,130,141,143,144,151,155^. |
|  | Percent agreement | - Proportion of observations for which two runs assign the same classification^90^. |
|  | Conditional error probabilities | - Predicted probabilities that the screening decision made by Loon Lens 1.0 is incorrect, given specific conditions of the model output, particularly the confidence level assigned to the citation and the tool’s inclusion or exclusion decision^72^. |
|  | Loss (L2) | - Quantifies the deviation of the current sensitivity and specificity estimates from their final (true) values as screening progresses. The loss is computed as the Euclidean distance between the estimated and true pairs of sensitivity and specificity. Lower values indicate that the current performance estimates are closer to the stable, final values, reflecting greater reliability at a given level of screening effort. $L_{2}\left( \hat{u},\hat{v} \right)=\sqrt{{(\mu-\hat{\mu})}^{2}+{(v-\hat{v})}^{2}}.$That is, given a true sensitivity/specificity (μ, ν), then for any estimate (ˆμ, ˆν)^169^. |
| Informativeness | Negative likelihood ratio | - The probability of a relevant reference failing AI screening divided by the probability of an irrelevant reference failing AI screening (1-sensitivity)/specificity^36,122,144^. |
|  | Positive likelihood ratio | - The probability of a relevant reference passing AI screening divided by the probability of an irrelevant reference passing AI screening, sensitivity/(1-specificity) ^36,122,144^. |
| Prediction | Precision | - The proportion of citations correctly predicted as relevant by the tool amongst all citations predicted as relevant by the tool (both correct and incorrect) ^16,29,31,32,38,40,41,43–45,49,52,53,56–58,63,66,68,70,72,75,78,79,83,84,86,87,92,95,102,107,111,115,117,119,124,126,129,134,147,149,151,161–163,166,172,175–177^ . - The percentage of citations judged to be relevant by human reviewer that had been predicted to be relevant by the tool^130^. |
|  | Positive predictive value (PPV) | - The proportion of relevant articles among articles that passed AI screening, TP/(TP+FP) ^31,34–36,39,44,54,71,74,77,109,122,127,138,144,165^. |
|  | Negative predictive value (NPV) | - The proportion of irrelevant articles among articles that failed AI screening, TN/(TN+FN) ^31,34,36,39,44,53,54,71,72,74,77,83,109,122,124,127^. - Negative predictive value was calculated by the formula: false positives ÷ (false positives + false negatives) ^35,144,165^. |
